# Deep sequencing of wheat sRNA transcriptome reveals distinct temporal expression pattern of miRNAs in response to heat, light and UV

**DOI:** 10.1038/srep39373

**Published:** 2016-12-22

**Authors:** Raja Ragupathy, Sridhar Ravichandran, Md. Safiur Rahman Mahdi, Douglas Huang, Elsa Reimer, Michael Domaratzki, Sylvie Cloutier

**Affiliations:** 1 Plant Science Department, University of Manitoba, Winnipeg, Manitoba, Canada; 2 Agriculture and Agri-Food Canada, Ottawa Research and Development Centre, Ottawa, Ontario, Canada; 3 Department of Computer Science, University of Manitoba, Winnipeg, Manitoba, Canada; 4 Agriculture and Agri-Food Canada, Morden Research and Development Centre, Morden, Manitoba, Canada

## Abstract

Understanding of plant adaptation to abiotic stresses has implications in plant breeding, especially in the context of climate change. MicroRNAs (miRNAs) and short interfering RNAs play a crucial role in gene regulation. Here, wheat plants were exposed to one of the following stresses: continuous light, heat or ultraviolet radiations over five consecutive days and leaf tissues from three biological replicates were harvested at 0, 1, 2, 3, 7 and 10 days after treatment (DAT). A total of 72 small RNA libraries were sequenced on the Illumina platform generating ~524 million reads corresponding to ~129 million distinct tags from which 232 conserved miRNAs were identified. The expression levels of 1, 2 and 79 miRNAs were affected by ultraviolet radiation, continuous light and heat, respectively. Approximately 55% of the differentially expressed miRNAs were downregulated at 0 and 1 DAT including miR398, miR528 and miR156 that control mRNAs involved in activation of signal transduction pathways and flowering. Other putative targets included histone variants and methyltransferases. These results suggest a temporal miRNA-guided post-transcriptional regulation that enables wheat to respond to abiotic stresses, particularly heat. Designing novel wheat breeding strategies such as regulatory gene-based marker assisted selection depends on accurate identification of stress induced miRNAs.

Plant speciation and long term adaptation to specific ecological niches involve genetic mutations and selection^1^. However, rapid adaptation to sudden exposure to abiotic stresses entails regulation of gene expression. In general, plants adapt to stress conditions by inducing an array of stress-responsive genes that are precisely regulated at the transcriptional, post-transcriptional and post-translational levels. Plant adaptation to environmental stress begins with its perception by sensory kinases followed by activation of signal transduction pathways culminating in expression modulation of regulatory as well as structural genes. Stress perception induces rapid accumulation of reactive oxygen species (ROS) and changes in cytosolic calcium (Ca^2+^)^2^. ROS and Ca^2+^ oscillation operate as secondary messengers to activate signal transduction pathways such as mitogen-activated protein kinases (MAPKs)^3^,^4^. Upon activation, MAPKs are imported into the nucleus where they phosphorylate stress-specific regulatory components such as transcription factors that modulate gene expression^5^,^6^. The products of these stress-responsive genes control several physiological processes such as stomatal closure and synthesis of hormones and metabolites (reviewed in ref. 7). In recent years, regulatory small noncoding RNAs (snRNAs) such as short interfering RNAs (siRNAs) and microRNAs (miRNAs) have emerged as key regulators of gene expression at both transcriptional and post-transcriptional levels^8^. Small RNAs (sRNAs), which are approximately 18–24 nt long, are classified based on differences in their biogenesis and function. Small RNAs derived from single stranded precursors with hairpin secondary structures are categorized as miRNAs while those derived from long double-stranded RNA (dsRNA) precursors belonged to the siRNA class^9^. MiRNA loci, similar to protein coding genes, are transcribed by RNA polymerase II (Pol II) which generate primary-miRNAs that fold into characteristic self-complementary stem-loop secondary structures that are cleaved by DICER-LIKE1 (DCL1) endonucleases to yield precursor-miRNAs which are further processed to release miRNA/miRNA* duplexes^10^. Mature miRNAs operate in post-transcriptional gene regulation by guiding mRNA cleavage and through translational inhibition^8^,^9^. SiRNAs are transcribed by RNA polymerase IV (Pol IV) from repetitive regions including transposable elements. The RNA-dependent RNA polymerase II (RDR II) catalyzes the synthesis of dsRNA which is cleaved by DCL3 to produce the siRNA duplex^11^. SiRNAs guide sequence-specific transcriptional gene silencing by recruiting methyltransferases to direct cytosine DNA methylation, to affect chromatin structure and to maintain genome integrity (reviewed in ref. 12). MiRNA-directed gene regulation is critical in several developmental processes including organ development^13^, hormone regulation^14^, nutrient homeostasis^15^, flowering^16^, stress response^17^ and DNA methylation^18^. Alteration of the expression level of stress responsive miRNAs and their targets has the potential to improve stress tolerance in crops^17^,^19^,^20^.

Recent changes in climatic condition have posed several unprecedented challenges to global food production. Optimal growth temperature is increasingly disrupted by extreme heat spells resulting in an estimated yield reduction of 6% for every degree Celsius^21^. In addition to increase in global temperatures, stratospheric ozone layer depletion at the poles (beyond 30° North and South latitudes) led to increased amount of UV radiation (280–320 nm) reaching the earth. Similarly, high intensity of solar radiation near the equator also contributes to higher UV levels (earthobservatory.nasa.gov). However, studies on small RNA transcriptome response of plants exposed to UV stress are limited, though UV exposure is found to have negative effects at the whole plant level^22^.

Plants grown under continuous light were a component of studies focused on understanding circadian rhythms. Recently, continuous light has been used under greenhouse conditions to increase production of horticultural crops, and a variant of the chlorophyll a/b binding protein 13-CAB13 gene conferring tolerance to continuous light has been identified in tomato^23^. In wheat, a novel strategy called speed-breeding is being adopted to accelerate genetic gain per year by employing continuous light under greenhouse conditions (http://hickeylab.com/our-projects/speed-breeding/; http://www.globalrust.org/poster-abstract-keywords/speed-breeding). However, transcriptomic studies profiling the consequence of this stress on molecular level processes are currently non-existent in the literature.

Recent advances in deep sequencing have enabled the identification of several miRNAs from various plant species and stress conditions^24^. Cross-talk among stress response signal cascade pathways has been reported in studies involving several abiotic stresses such as heat, cold, drought and salinity^25^. Hence, we hypothesize that small RNAs associated with stress responses may share transcriptional regulatory networks. In addition, monitoring the temporal expression patterns of miRNAs in response to abiotic stresses is fundamental to gain insights into their functions. Previous studies have shown that differential expression of certain miRNAs are specific to growth and stress conditions^16^,^17^. Hence, understanding plant response to various stresses over several time points is essential to design appropriate breeding strategies. With this objective in mind, we exposed wheat (*Triticum aestivum* L.) plants to heat, light and ultraviolet radiation (UV) stresses and analyzed their miRNA expression profile at 0, 1, 2, 3, 7 and 10 days after treatment (DAT) using a replicated experimental design and the Illumina high-throughput sequencing platform.

## Results

### Sequencing and sRNA profile

A total of 72 sRNA libraries corresponding to the four treatments (control, heat, continuous light and UV), three biological replicates and six sampling time points (0, 1, 2, 3, 7 and 10 DAT) was constructed and their sequencing on the Illumina platform generated 619 M raw reads. An overview of the sequence processing is illustrated in Fig. S1 and detailed statistics for each library can be found in Table S1. After sequence quality check with FASTQC and extraction of the 18–24 bp sequences using Geneious, the 524 M reads that remained were grouped into a non-redundant dataset of 129 M distinct tags. After removal of other non-coding RNA and chloroplast sequences, we obtained 121 M distinct tags corresponding to 427 M raw reads. The distinct tags were consistently named across libraries and a total of 15,158 distinct tags corresponding to 294 M raw reads were retained on the basis of their read count exceeding ten reads per million (RPM) in at least one library. A total of 232 distinct tags represented by 14 M reads were accurately annotated with evidence for biosynthesis based on their mapping to previously annotated precursor coding loci from the IWGSC draft assembly^26^ and/or Kurtoglu *et al*.^27^, and those that match to previously annotated miRNAs from Sun *et al*.^28^. For 186 out of the 232 miRNAs, we found evidence of expression of their complementary star miRNA sequence with perfect 2-bp overhang between mature and star sequences on the 3′ strand of the stem-loop secondary structure (Table S2).

**Figure 1:**
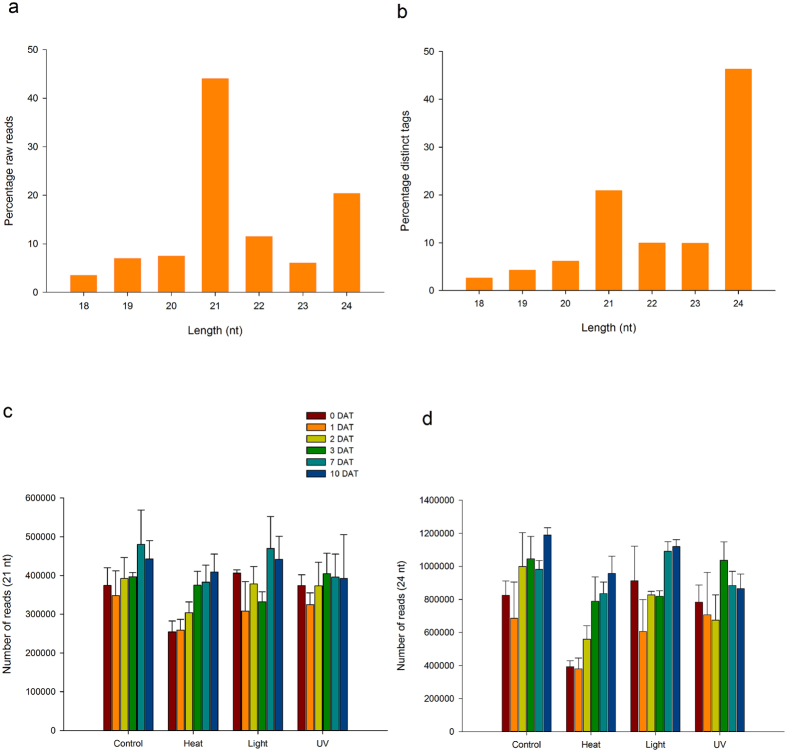
Overview of small RNA profile. (**a**) Size distribution of raw reads after size selection with Geneious. (**b**) Size distribution of distinct tags prior to removal of ncRNA and chloroplast sequences. (**c**) Distribution of 21 nt-long distinct tags by treatment. (**d**) Distribution of 24 nt-long distinct tags by treatment. The bars represent the mean and standard deviation from three biological replicates.

The size distribution of total reads and distinct tags was assessed (Fig. 1). The 21 nt size class was the most abundant raw read class representing 44% of the total read count (Fig. 1a) but these sRNAs were grouped into fewer distinct tags than the 24 nt size class (Fig. 1b). The total number of high quality18–24 nt raw reads and distinct tags was similar in control, light and UV stressed plants but was 15–20% less in heat-stressed plants on average across all time points (Fig. S2a,b). This was more acute for the early time point libraries where the numbers of 21 and 24 nt distinct tags were reduced by 22–32% and 67–77%, respectively, in heat-stressed plants at 0, 1 and 2 DAT while being comparable to the other treatments at 3, 7 and 10 DAT (Fig. 1c,d). Raw data and read count files were deposited in the short read archive (SRA) of GenBank under accession number GSE83738.

### Identification of conserved miRNAs expressed in response to heat, light and UV stresses

**Figure 2:**
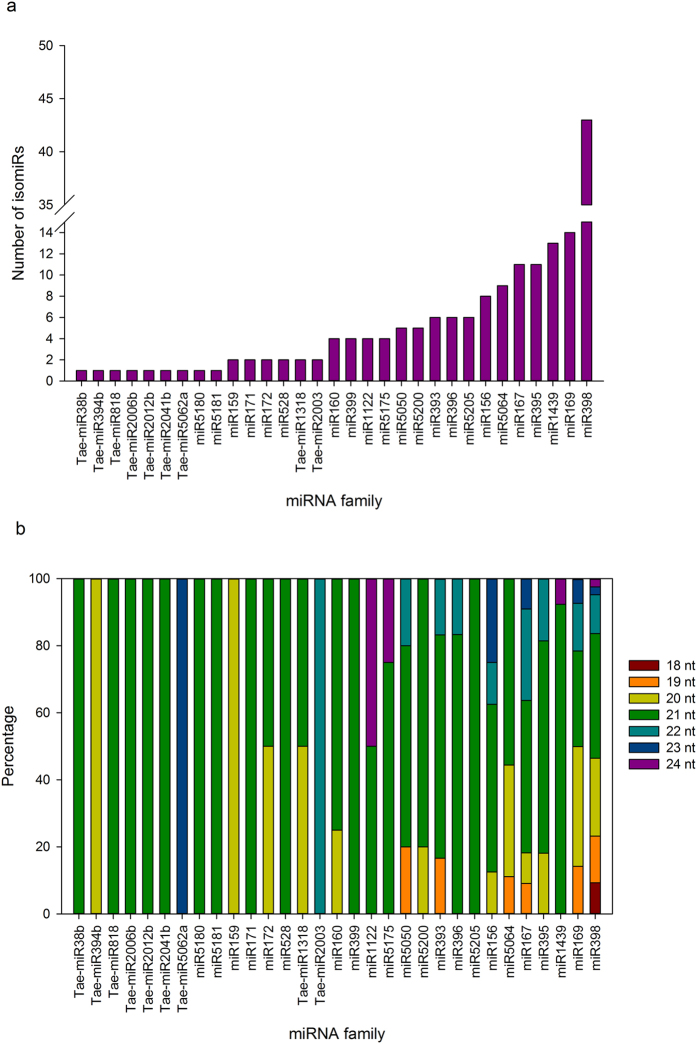
Conserved miRNA families. (**a**) Distribution of family members and isomiRs assigned to a subset of 31 mature miRNA families with distinct unambiguous precursor evidence. (**b**) Relative abundance of different size classes of the same 31 mature miRNA families.

Alignment of the 15,158 distinct tags (≥10 RPM in at least one library) to previously annotated wheat precursors revealed 232 conserved miRNAs belonging to 35 distinct miRNA families, among which, 174 members belonging to 31 families were found to originate from individual precursors, whereas 58 members were found to be associated with precursors which could not be unambiguously assigned to distinct mature miRNA families (Table S2). The distinct tags that perfectly matched previously known miRNAs with precursor sequences were represented by 14.9 M reads, i.e., only 2.4% of the total reads (Fig. S1) and nearly 63% of the conserved miRNAs were 21-mers (Fig. S3). Only nine families were represented by a single tag; the other 22 were represented by two to 43 distinct tags comprising paralogs and isomiRs (Fig. 2a). Of the four ambiguous families, all members were 21-mers (Table S2). Families miR167, miR169 and miR398 were represented by variants of five to seven of the length between 18 to 24-mers while miRNAs from miR399 and miR5205 were all 21 nt-long (Fig. 2b).

### Differential expression analysis reveals a distinct expression pattern in heat stressed plants

The total read count for each miRNA varied from 0 to 471,026, normalized to 0 to 602,765 RPM, depending on the library (Table S3). Their expression levels also varied greatly even among members of a family and/or isomiRs. For example, miR398_tgtgttcccagctcgaccccg was observed between 0 and 842 RPM across libraries while the expression of miR398_agggtcgaactgagaacacat reached 16,832 to 602,765 RPM (Table S3).

**Figure 3:**
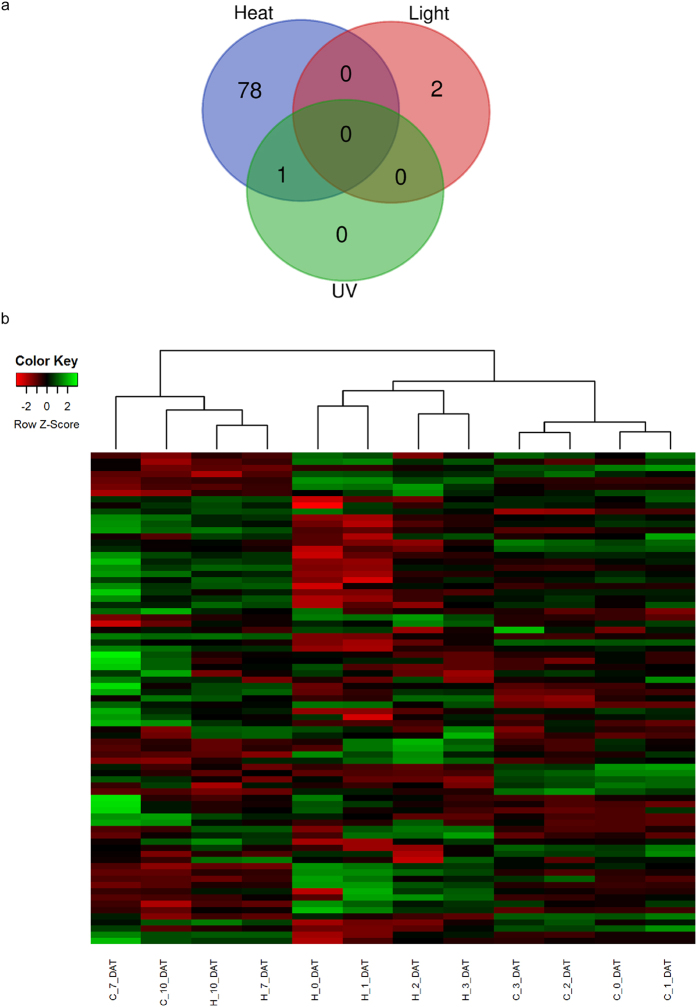
Differential expression of miRNAs following stress treatments. (**a**) Venn diagram representing the number of the miRNAs differentially expressed in response to heat, light and UV stresses. (**b**) Heat map of differentially expressed miRNAs in response to heat stress. The green color represents over expressed miRNAs while red is for under expressed miRNAs. The dendrogram at the top represents the clustering of the control (C) and heat (H) treatments at the six sampling time points of 0, 1, 2, 3, 7 and 10 days after treatment (DAT) labeled at the bottom.

Seventy-nine of the 232 miRNAs were differentially expressed (FDR < 0.05) upon heat stress but only two and one were so following continuous light and UV stresses, respectively (Fig. 3a). Interestingly, 46 and 44 of the 79 miRNAs were down regulated at 0 and 1 day after heat stress, respectively (Fig. S4). A heat map and cluster dendrogram of differentially expressed miRNAs revealed the temporal dynamics at the different sampling time points after heat stress (Fig. 3b). The expression profile of control plants at 0, 1, 2 and 3 DAT formed a distinct cluster from the heat-stressed plants at the same sampling time points, while they clustered together at 7 and 10 DAT, indicating that response to heat stress was attenuated from one week after the end of the stress onward (Fig. 3b). Expression profiles of miR398, miR156 and miR169 family members and isomiRs all showed reduced expression in the early recovery period after heat stress (Fig. S5a–c). In contrast, expression of miR167 and miR396 isomiRs was induced by heat stress (Fig. S5d,e), while expression of three of the four miR399 isomiRs was upregulated from 3 to 10 DAT (Fig. S5f).

### Prediction of miRNA targets and construction of regulatory networks

**Figure 4:**
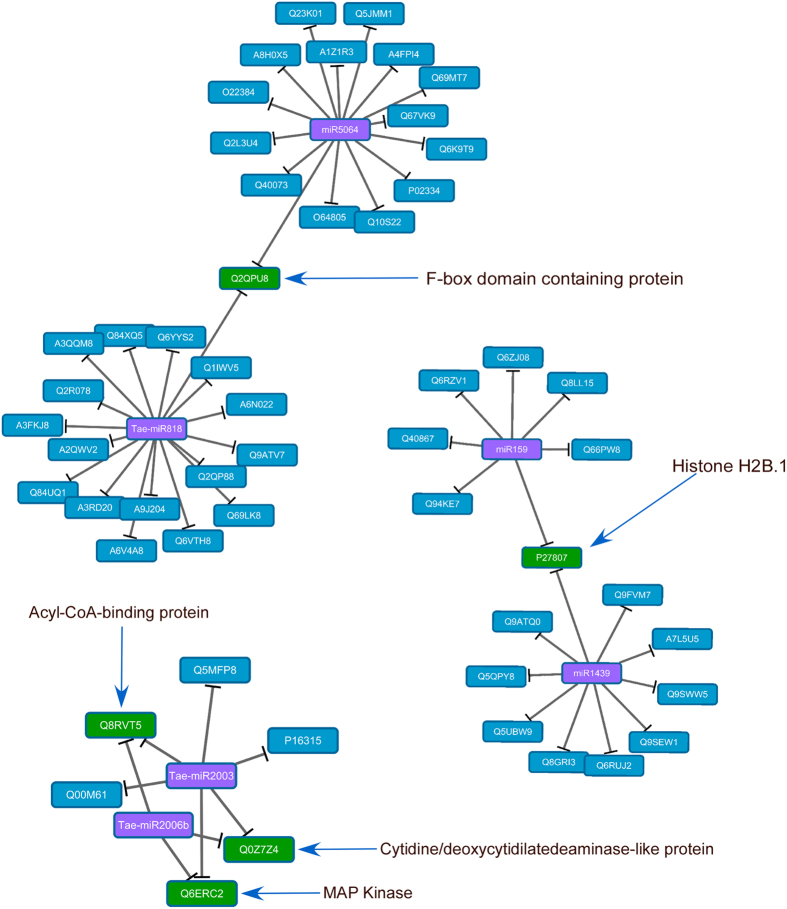
Three examples of Cytoscape networks of differentially expressed miRNAs and their targets. MiRNAs are in purple boxes in the middle of the networks and their targets are identified by their UniProtKB ID in the surrounding blue boxes. The green boxes identify the mRNAs predicted to be targeted by more than one miRNA. Targets addressed in the discussion are marked with blue arrows.

**Figure 5:**
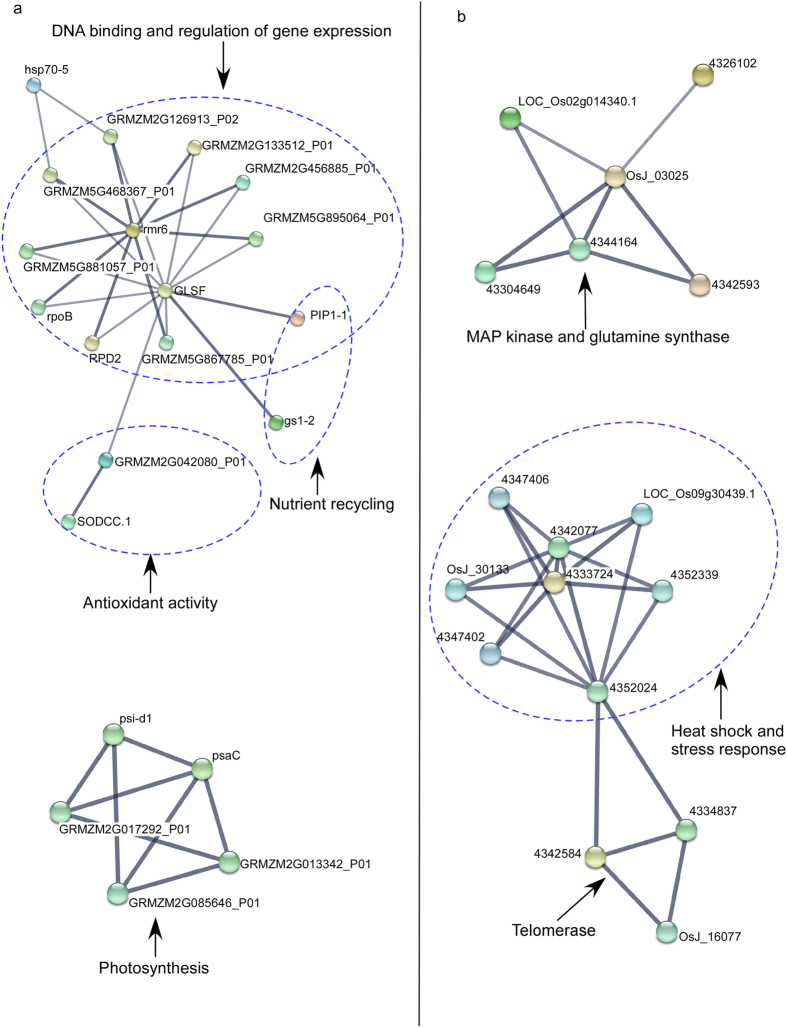
STRING network view of known and predicted interaction partners. The miRNA targets were used as query to search previously annotated proteins. (**a**) Maize (*Zea mays*), and (**b**) Rice (*Oryza sativa*). The confidence view screen shows the known and predicted protein-protein interactions where stronger associations are represented by thicker lines.

Using psRNA target, 70 of the differentially expressed miRNAs were predicted to target 44 putative mRNAs of the wheat unigene set and 60 from other plant species (Table 1). Gene ontology (GO) annotation showed that 18 of the targets were involved in nucleic acid binding, six were associated with kinase activity, 26 were involved in ion binding activity and another six with oxidoreductase activity (Table S4). A detailed analysis indicated that miRNAs downregulated in response to heat stress, such as miR156, Tae-miR818 and miR169, primarily targeted transcripts associated with activation of signal transduction pathways and transcription factors such as receptor-like kinases, CCAAT and MIKC-type MADS-box transcription factors. MiR169 was predicted to specifically target three members of the CCAAT-box transcription factor complex (Table 1). Other classes of targets included squamosa promoter-binding-like (SPL) proteins and flowering locus-T (FT) which mediate flower development and superoxide dismutase (SOD) which is involved in oxidative stress responses. MiRNAs induced upon heat stress at different time points, such as miR1439 and miR159, were primarily associated with histone variant H2B.1 and signal transducers including MAP and LRK14-Ser/Thr kinases. MiR160 targeted stress responsive genes such as auxin response factors and drought-responsive factors, while MiR167 and miR396 are known to target signal transducers and nutrient reservoir activity, respectively. MAPKs, F-box family proteins, histone H2B.1, acyl-CoA-binding proteins and cytidine/deoxycytidylate deaminase-like proteins were targeted by more than one miRNA (Fig. 4). Certain classes of kinases and enzymes involved in oxidative stress, such as SOD and peroxidases, were identified as targets of miRNAs that were both suppressed and induced in response to heat stress. A search for potential interacting proteins using annotated proteins in maize (*Zea mays* L.) showed that proteins associated with regulation of transcription (histones and DNA-dependent RNA polymerases), antioxidant activity, nutrient recycling and photosynthesis clustered together (Fig. 5a). A similar search with annotated proteins in rice (*Oryza sativa* spp. *Japonica* L.) revealed distinct clusters with telomerase, heat shock, glutamine synthase and glucosyl transferase associated proteins (Fig. 5b). Protein names and description of potential interacting proteins are listed (Tables S5 and S6).

## Discussion

MiRNA-guided post-transcriptional regulation has emerged as an essential component in numerous developmental processes and in stress responses in plants since their first discovery in the nematode *Caenorhabditis elegans*^29^. The advent of next generation sequencing has enhanced the identification of miRNAs across plant species, including those whose genome has not been fully sequenced^30^ (http://www.mirbase.org/cgi-bin/browse.pl). Recent studies have indicated that a set of miRNA families that regulate development related transcription factors are highly conserved and have similar targets across land plants including ferns, gymnosperms and angiosperms, spanning an evolutionary period of ~480 million years^31^. Therefore, a thorough understanding of miRNA-guided regulation underlying rapid stress adaptation can offer alternate strategies to develop stress tolerant crops in the context of unpredictable weather associated with climate change through artificial miRNA-guided gene silencing^32^ or the application of DNA markers developed based on precursor coding loci^33^. In this study, we report the identification of 232 genuine wheat miRNAs belonging to 38 families annotated with strong evidence of precursor sequence, presence of cognate star miRNA sequence and supporting stem-loop structure with 2-nt offset between mature and star miRNA reads, as per the stringent guidelines for miRNA^34^ and precursor^35^ annotations. Our results indicated that heat but not continuous light and UV stresses altered the expression profile of a significant number of miRNAs. We demonstrated that the accumulation of miRNAs in response to heat stress was highly regulated in a temporal manner to fine-tune gene expression. The miRNA targets included several mRNAs whose proteins have been previously shown to mediate stress tolerance in plants^17^,^19^,^30^.

Only 2.4% of the total reads we generated represented miRNAs, a substantially smaller fraction than the ~9% reported in Arabidopsis exposed to stresses such as dehydration, salinity, cold and abscisic acid^36^. Three factors may have contributed to this difference. First, our annotation, based on the 98,068 precursor coding loci of the IWGSC draft assembly^26^, is likely an under estimation of the actual number of miRNA loci as a consequence of the gaps remaining in this original assembly. Second, application of stringent criteria such as the requirement of a minimum of 10 RPM combined with precursor, star sequence and ideal hairpin supports for declaring miRNA excluded putative novel miRNAs without precursor support and/or those expressed at low levels in favor of fewer but more genuine miRNAs. We opted for the identification of fewer genuine miRNAs as opposed to a larger number that would likely have contained false positives. Third, the IWGSC draft assembly^26^ excluded short contigs of <200 bp matching the Triticeae Repeat Database (http://wheat.pw.usda.gov/ITMI/Repeats) and, as a consequence, precluded the identification of likely most of the genuine transposon-derived precursor loci such as MITE-derived precursor loci^37^.

Our results indicated that the 21 nt class was the predominant fraction among sRNA raw reads while the 24 nt class prevailed after assembly into distinct tags. The predominance of these size classes is a typical outcome of cleavage by DCL endonucleases, namely DCL1 for miRNAs and DCL3 for siRNAs^38^. The 21 nt sRNAs are mostly miRNAs assembled into distinct reads based on the diversity of the members of each family. The 24 nt sRNAs are mostly siRNAs whose relatively higher proportion of distinct tags reflects their origin from transposons and repetitive elements which account for ~80% of the wheat genome^26^. The presence of thousands of transposon families in the wheat genome has the potential to give rise to an enormous diversity of the 24 nt class siRNAs. Pol IV transcripts from transposons were recently discovered to generate the actual precursors of the 24 nt class of siRNAs^39^,^40^. Considering that a single precursor transcript of 24–45 nt long^39^ or 30–40 nt long^40^ is sufficient to create a unique 24 nt tag, a single transposable element spanning several kilobases has the potential to give rise to several precursor transcripts. Although only a minority of miRNAs belongs to the 24 nt class, some share a function similar to siRNAs. Indeed, a DCL3-dependent 24 nt variant miRNA has been identified to direct DNA methylation similar to siRNA^18^.

A recent study showed that even if miRNA size classes *per se* may depend on the taxonomic lineage, the 21 nt size class remained the most predominant size class among 31 species representing a wide range of vascular plants^31^. Specific family enrichment in some species/lineages may account for the size class diversity of some lineages. For example, miR156 and miR168, enriched in some species, exist predominantly as 20 and 21-mers, whereas miR472, miR482 and miR2118, enriched in others, are 22-mers^31^. Interestingly, some miRNA size classes seem to display distinctive features. For example, the less abundant 22 nt miRNAs were previously shown to arise from fold-back precursors that carry asymmetric bulges^41^.

Members of a miRNA family can display sequence and length variants^42^. They can be encoded by different loci (for instance, MIR159a and MIR159b), paralogs (for instance, MIR159a-1 and MIR159a-2) or isomiRs (length variants generated from a single precursor by DCL and/or sequence variants arising out of post-RNA editing). Accurate identification and consequently nomenclature to distinguish family members originating from paralogs encoded by distinct loci or isomiRs remains challenging because this field of research is in its infancy^43^. In our experiment, the number of members of the mature miRNA families and their length variants were given in the Fig. 2 but the lack of a complete genome sequence for wheat precluded us from obtaining full comprehension of the origin of each family members. In general, the actual number of miRNA coding loci varies for individual families and depends on the species. For instance, 14, 17, 18, 25 and 32 coding loci were identified for miR169 in *Arabidopsis, Oryza, Zea, Vitis* and *Populus*, respectively^44^.

The overall lower abundance of sRNAs in heat-stressed plants reflects the down regulation of some heat stress-associated miRNAs, particularly in the early recovery period. The consistent reduction in the overall miRNA counts correlated with the expression profiles where the only stress that significantly altered the differential expression of miRNAs was heat. Similar reduction in sRNA read counts in the range of 20–35% have been reported in rice exposed to drought, cold and salt stresses^45^ and in poplar exposed to drought^46^. Light and UV stresses had previously been demonstrated to alter sRNA expression^47^,^48^,^49^. However, important differences exist between our experiment and these, particularly as they pertain to the tissue exposed to the stress and the time period during which the sampling was performed. For example, in Arabidopsis^47^,^49^ and wheat^48^, young seedlings were subjected to stress and the plant tissues were sampled between 2 and 24 h post-treatment. Hence, we speculate that wheat plants at the boot stage were relatively resilient to UV and light stresses, at least at the levels and intensities of our experimental conditions, as neither of these stresses significantly altered the expression level of miRNAs, even immediately at the end of the stress period, i.e., 0 DAT. Alternatively, the intensity and/or the duration of the light and UV stresses applied herein may have been insufficient to trigger an miRNA response in wheat leaves at the boot stage.

The abundance of distinct tags ≥10 RPM was similar across libraries, suggesting that the miRNA transcripts were tightly controlled at the transcriptional level through the regulation of their canonical or non-canonical biosynthetic pathways or as a consequence of the turnover of mature miRNAs^50^. Notably, ~99% of distinct tags were expressed at less than 10 RPM. This means that the majority of the sRNA was discarded because of their low level expression, yet subtle expression differences may still have potential impact in stress adaptation. The highly expressed miRNAs, considered conserved in plants^31^, are hypothesized to regulate cellular and developmental pathways. MiRNAs expressed at low levels are deemed non-conserved and lineage-specific regulators of stress response.

Although deep sequencing is a valuable tool for the identification and expression profiling of miRNAs, factors such as genotype, growth conditions, developmental stage, stress duration and intensity, sampling size and time, and sequencing depth can all affect the relative abundance of miRNAs^24^. In a previous study based on 12-day old seedlings of heat tolerant cultivar HD2985 subjected to 42 °C for 2 hours, 44 mature known wheat miRNAs (miRbase v19) were identified from mixed samples of root, stem, flag leaf and pollen tissues, among which, 19 were differentially expressed including four families (miR1130, miR1136, miR395a and miR408) showing expression only in heat stressed plants^51^. Inspite of differences between our studies in terms of genotypes (Glenlea vs HD2985), duration of heat stress exposure (5 days vs 2 hours), stage of sampling (boot stage vs seedling), nature of samples (leaf vs mixed), precursor evidence (~98,000 from the IWGSC draft assembly vs wheat ESTs) and method of differential expression analysis (edgeR vs log2 ratio), five major miRNA families (miR156, miR159, miR160, miR167 and miR398) associated with proteins of known heat signaling pathways were differentially expressed in both studies.

As miRNA levels are dynamically altered, especially in response to stress, quantifying miRNAs at fewer time points may limit our understanding of the role of individual miRNAs^52^. The abundance of miRNAs varied temporally during the recovery period that followed the heat stress suggesting a precise post-transcriptional regulation. For example, miR528 (Fig. S6a) and miR398 were down regulated in response to heat stress at 0 and 1 DAT. Consequently, their putative SOD targets were likely to accumulate at higher levels, enhancing antioxidant defense. The accumulation of SOD catalyses the conversion of ROS such as superoxide to peroxide which is subsequently detoxified to oxygen and water by catalases and peroxidases^53^. MiR398 was demonstrated to be downregulated in response to oxidative stress in order to control Cu/Zn superoxide dismutase (CSD1 and CSD2) in Arabidopsis^17^. Similarly, miR398 suppression in response to drought stress alters peroxidases activity in maize^54^. ROS can be a toxic intermediate but it can also be a key signalling molecule (reviewed in ref. 53). ROS are also produced under normal metabolic processes and their role in signalling depends on their synthesis level and that of antioxidant enzymes. Response to heat stress may thus be mediated by antioxidant enzymes such as SOD and peroxidases whose production would be eased through the down regulation of specific miRNAs.

MiRNAs associated with flowering were also suppressed in response to heat stress. MiR156, referred to as a count-down timer necessary for sustaining the juvenile phase^55^, targets a SPL family, which in turn, promotes the transition from the juvenile to the adult phases. Hence, suppression of miR156 under heat stress accelerates flowering which makes evolutionary sense^16^. Also, miR396 and miR167 are involved in physiological adaptation including stomatal movement and cell division. MiR396 targets growth regulating factors and mediates cell division and differentiation^20^. Moreover, transgenic plants overexpressing miR396 had lower stomatal density and displayed improved tolerance to drought^19^. On the other hand, increased miR167 levels and consequent reduction of its auxin response factor target impaired male and female fertility^56^. Repression of auxin signals also enhances disease tolerance to bacterial pathogens^57^. Interestingly, miR396 and miR167 expression levels were induced with heat stress but were similar to control plants from day 7 onward. Sampling time plays a crucial role in understanding the function of stress responsive miRNAs because their expression is modulated during development. Hence, a critical understanding of the control of miRNA biogenesis and turnover is required to strategize their use for the development of stress tolerant crops because constitutive expression of miRNAs may have a fitness cost.

In wheat, selective gene loss and independent evolution of genes associated with abiotic stress tolerance occur within different genomes and sub-genomes^26^. For example, transcription factors are encoded by multiple loci^58^ and control the expression of several genes in qualitative and quantitative manners. Though miRNAs targeting transcription factors were reported, it is difficult to ascribe a function to miRNAs that target transcription factors because the latter can control the expression of multiple genes and the overall outcome may lead to functional redundancies. Also, miRNAs targeting MAPK activity were functionally characterized in several plant species (reviewed in ref. 59) but few have been functionally characterized in wheat^60^. Hence, further validation of the role(s) of miRNA targeted kinases and transcription factors is required because these enzymes activate multiple signalling pathways and control the expression of multiple genes involved in stress tolerance.

Small RNA mediated histone modifications, methylation of DNA sequences and chromatin remodelling can affect the expression of stress responsive genes. Common modifications of histones include phosphorylation, ubiquitination, acetylation and methylation^61^. Histone modification plays a central role in transgenerational inheritance where memory of stress exposure is retained and imprinted in the subsequent generation^62^. Heat-stress induced the co-expression of miR159 and miR1439 (Fig. S6g,h) that were predicted to target histone H2B.1, a core component of the nucleosome. Although there are currently no evidence for the role of histone H2B.1 in stress tolerance, another histone variant, namely H2A.Z, was found to be a factor in thermosensing in Arabidopsis^63^. The role of methyltransferases in chromatin modification and transgenerational inheritance is emerging in both animals and plants^64^,^65^. MiR395, predicted to target methyltransferases, was not differentially expressed upon any of the stresses across all time points in our experiment. This may be due to the tissue and stage sampled, i.e. leaves at the pre-reproductive stage.

MiRNAs act as mobile signals to regulate post-transcriptional gene silencing and guide epigenetic modifications^66^ by targeting ~35 proteins involved in the RNA-dependent DNA methylation (RdDM) pathway^12^. Differential expression of miRNAs from plants exposed to abiotic stresses hints at their mode of action in stress tolerance and/or stress avoidance inferred based on their interactions with target transcripts including transcription factors. Hence, further validation of miRNA targets remain an essential step towards designing translational strategies to capitalize on these regulatory genes as tools such as DNA markers for the genetic improvement of wheat.

We believe the results presented herein to be at the forefront of wheat and even plant miRNA research. We would like to emphasize a number of attributes of our experiment that are rarely observed in this type of research by themselves, let alone in combination. These are (1) the simultaneous study of multiple abiotic stresses, (2) the use of a time series, (3) the application of true biological replications, (4) the utmost strictness of the criteria used for the identification of the genuine miRNAs and (5) the large number of the latter, i.e., 232. While much remains to be done in wheat genomics and epigenetics, we hope that the high standards applied throughout the execution of this experiment and its data analyses render the results more readily applicable for crop improvement.

## Methods

### Plant material and growth conditions

Wheat (*Triticum aestivum* L.) cv Glenlea plants, obtained from a single self-fertilized homozygous line, were grown in a PG-40 growth chamber (Conviron Technologies, Winnipeg, Canada) under long-day conditions of 16 h light (300 μmol m^−2^ s^−1^) at 18 °C and 8 h darkness at 16 °C. At the boot stage, three sets of 24 plants each were exposed to one of three stress conditions for five consecutive days. The first set was exposed to continuous light. The second set was exposed to UV light for two minutes every day of the 5-day period using a 30 W TUV T8 bulb (Philips) producing short-wave UV radiation with peak at 253.7 nm (UVC). The third set, called heat-stressed, was transferred to a growth chambers where the temperature was kept constant at 37 °C day and night. These plants were well watered twice per day during the 5-day stress exposure to avoid a confounding drought stress. The remaining 24 control plants were kept in the growth chamber at 16 h/8 h day/night at 18/16 °C. After the 5-day stress exposure treatment, all plants were returned to the original growth chamber under control conditions. The experiment was conducted as a completely randomized block design with three biological replicates where each treatment and replicate corresponded to a group of eight plants.

### Sampling and RNA extraction

Leaf tissue was collected from individual plants at six time points: 0, 1, 2, 3, 7 and 10 DAT where 0 DAT corresponded to tissue sampled immediately at the end of the 5-day stress exposure period, prior to their return to the non-stressful growth conditions. Leaf tissue pieces, approximately 2.5 cm in length, were pooled from the eight plants that represented each treatment and replicate. The harvested leaf tissue pools were flash-frozen and stored in liquid nitrogen until RNA extraction. For the latter, tissue samples were ground to a fine powder with liquid nitrogen in a mortar and pestle and total RNA was isolated from homogenized tissue samples using TRI-reagent as per manufacturer’s instructions (Ambion, Naugatuck, CT). Total RNA quality and quantity were assessed on an Agilent 2100 Bioanalyzer with the RNA 6000 Nano chip (Agilent Technologies, Santa Clara, CA). A total of 72 high-quality total RNA samples, corresponding to three biological replicates, four treatments and six sampling time points, were produced.

### Small RNA library construction and sequencing

Small RNA libraries were constructed for each of the 72 samples from 5 μg of total RNA using a plate-based method developed at the BC Cancer Agency Genome Sciences Centre (Vancouver, BC, Canada). Briefly, total RNA samples were mixed with oligo-dT microbeads and loaded into a 96-well MACS column (Miltenyi Biotec, Germany). The sRNA fractions were recovered from the flow-through and precipitated with ethanol. Quality was assessed for a subset of 12 samples using an Agilent Bioanalyzer RNA 6000 Nano chip (Agilent Technologies). An adenylated 3′-adapter (5′/5rApp/ATCTCGTATGCCGTCTTCTGCTTGT/3ddC/3′) was ligated using a truncated T4 RNA ligase (New England BioLabs, Ipswich, MA) by incubating at 22 °C for 1 hour. An RNA 5′-adapter (5′-GUUCAGAGUUCUACAGUCCGACGAUCUGGUCAA-3′) was then added using a T4 RNA ligase (Ambion) by incubating at 37 °C for 1 h. The first strand cDNA was synthesized using Superscript II reverse transcriptase (Invitrogen, Carlsbad, CA) and RT primer (5′-CAAGCAGAAGACGGCATACGAGAT-3′). The cDNA was used as template for PCR amplification where unique index sequences were introduced to enable identification of the pooled libraries. PCR cycling conditions were 98 °C for 30 sec, followed by 15 cycles at 98 °C for 15 sec, 62 °C for 30 sec and 72 °C for 15 sec and a final incubation at 72 °C for 5 minutes. The quality of the libraries was assessed using a Caliper LabChipGX DNA chip (PerkinElmer, Waltham, MA). Three pools of randomly assigned sRNA libraries were created, resolved on a gel and, the 145–160 bp fractions were size-selected. Pooled libraries were ethanol precipitated and quality checked using an Agilent Bioanalyzer DNA1000 chip (Agilent Technologies). Each pooled library was diluted for cluster generation on a HiSeq 2000 flow cell according to manufacturer’s instructions. Small RNA sequencing was performed using a 50 cycle HiSeq SBS v4 kit.

### Preprocessing of sequence data

A tool chain for processing of the data was constructed. First, reads were separated into individual samples based on their index sequences. Adapter sequences were trimmed using a custom Perl script and quality assessment was performed using FastQC (http://www.bioinformatics.babraham.ac.uk/projects/fastqc/). Reads ranging from 18 to 24 bp were filtered and exported as fasta files using Geneious (Biomatters Ltd, Auckland, New Zealand). Redundant reads from each dataset were grouped as distinct tags to calculate the read counts and were normalized as RPM to account for variation in sequencing depth across libraries. The non-redundant or distinct tags were used to query the Rfam^67^ and wheat chloroplast (http://www.ncbi.nlm.nih.gov/nuccore/NC_002762.1) databases. Sequences matching rRNAs, tRNAs, snRNAs, snoRNAs, other long non-coding RNAs and chloroplast sequences were removed using a python script (https://github.com/ECORC-Cloutier/mirna-pipeline). The remaining distinct tags that were identical across libraries were then consistently named.

### Identification of conserved miRNAs

Accurate miRNA annotation was carried out following guidelines of Meyers *et al*.^34^ and Kozomara and Griffiths-Jones^35^ for mature miRNA and precursor evidence, respectively. Distinct tags represented by ≥10 RPM in at least one library were mapped to the 98,068 miRNA coding loci previously identified from the hexaploid bread wheat assembly^26^ using Bowtie2^68^. Briefly, a fasta database was generated by extracting precursor sequences, distinct tags were aligned to the precursor sequences using Bowtie2 and tags with perfect match were retained. These tag sequences were then matched to another set of high confidence precursor sequences^27^ and only those with perfect matches were extracted for hairpin structure and star sequence prediction using RNAfold^69^ (https://www.tbi.univie.ac.at/RNA). Only the distinct tags that showed evidence of biosynthesis based on a matching precursor that could fold into secondary hairpin structure with <−0.2 kcal/mol/nt were kept^35^. In addition, presence of the complementary star sequence in the opposite strand with a 2 bp offset on the 3′-strand of the hairpin was assessed. Distinct tags were also matched to previously annotated miRNA and star sequences^28^. Distinct tags with strong evidence for their biosynthesis, including both precursor and star sequence supports were selected for differential expression analysis, miRNA target prediction and generation of gene regulatory networks.

### Differential expression analysis, miRNA target prediction and construction of transcriptional regulatory networks

The read counts of miRNAs annotated with strong evidence for biosynthesis from all 72 libraries (4 treatments × 3 replications × 6 time points) were used for differential expression analysis. The read counts were normalized^70^ to account for differences in library sequencing depth and RNA composition using edgeR^71^. A general linear model (GLM) was used to identify miRNAs differentially expressed across treatments and over time. MiRNAs with false discovery rate <0.05 were considered differentially expressed^72^. Heat maps were generated with normalized log2 read counts.

Putative miRNA targets were predicted from wheat unigenes (DFCI wheat gene index version 12) using psRNATarget^73^ with default parameters. The putative gene targets were identified using the UniProtKB database (http://www.uniprot.org/help/uniprotkb). Differentially expressed miRNAs and their putative target gene(s) and predicted protein(s) were used to generate regulatory networks underlying stress response. Putative target proteins and their associated GO terms and functions were identified using AgBase GORetriever (http://www.agbase.msstate.edu/cgi-bin/tools/goretriever_select.pl). Gene regulatory networks were constructed using Cytoscape^74^. MiRNA targets were used as query to search annotated protein entries in maize and rice using the online tool STRING v10 (http://string-db.org/) and potential interacting protein partners were predicted using a high confidence parameter of 0.7^75^.

## Additional Information

**How to cite this article:** Ragupathy, R. *et al*. Deep sequencing of wheat sRNA transcriptome reveals distinct temporal expression patterns of miRNAs in response to heat, light and UV. *Sci. Rep.* **6**, 39373; doi: 10.1038/srep39373 (2016).

**Publisher's note:** Springer Nature remains neutral with regard to jurisdictional claims in published maps and institutional affiliations.

## Supplementary Material

Supplementary Information

Supplementary Table S2

Supplementary Table S3

## Figures and Tables

**Table 1:** PsRNA targets predicted for miRNA families containing isomiRs differentially expressed following heat stress.

Mature miRNA family	No. of family members including isomiRs	No. of differentially expressed members	Predicted target protein classes	
			Wheat	Other plant species
miR398	43	29	A3FKE5 Cluster: Superoxide dismutase	P17816 Cluster: Glycine-rich cell wall structural protein precursor
			Q93YY0 Cluster: 68 kDa protein HP68	
			A7J2I2 Cluster: Plasma membrane intrinsic protein	
			Q41539 Cluster: Endochitinase precursor	
			Q9LKM4 Cluster: Cold-responsive protein	
			P00228 Cluster: Ferredoxin; chloroplast precursor	
			Q9S7U0 Cluster: Inositol-3-phosphate synthase	
miR156	8	7	Q9ATQ5 Cluster: LRK33 (ATP binding)	Q0JGI1 Cluster: Squamosa promoter-binding-like protein 2
				Q49I55 Cluster: Teosinte glume architecture 1
				A6MD03 Cluster: F-box family protein
				Q16FC2 Cluster: Cytochrome P450
				A0ZPR8 Cluster: Telomere binding protein
				A6MD03 Cluster: F-box family protein
				Q6EUS6 Cluster: Aspartate aminotransferase
				Q3IS37 Cluster: Glycosyl/glycerophosphate transferase
				Q2QQS5 Cluster: Cyclin-T1-4; n = 1; *Oryza sativa japonica*
miR169	14	6	Q6Q1B9 Cluster: CCAAT-box transcription factor complex WHAP12 - DNA binding	Unknown
			Q6Q1C5 Cluster: CCAAT-box transcription factor complex WHAP6 - DNA binding	
			AAS78483.1 CCAAT-box transcription factor complex WHAP9	
miR167	11	4	A7U1×4 Cluster: ABP-1	Unknown
			Q4VWF0 Cluster: Histidine-containing phosphotransfer protein - Signal transducer	
miR399	4	4	A7TVU8 Cluster: Asynapsis 1	A2Q2W7 Cluster: 4Fe-4S ferredoxin; iron-sulfur binding
				Q75HA3 Cluster: Expressed protein - Intergral membrane protein
				Q1III0 Cluster: ABC nitrate/sulfonate/bicarbonate transporter
				Q84VB8 Cluster: Nuclear matrix constituent-like protein
miR5050	5	4	A7LHB7 Cluster: Alpha gliadin - nutrient reservoir activity	Unknown
			Q3S2H9 Cluster: Wall-associated kinase-like 2	
miR396	6	3	Q03387 Cluster: Eukaryotic initiation factor iso-4F subunit p82-34 - regulation of translation	Unknown
			Q41583 Cluster: Initiation factor (Iso) 4f p82 subunit - regulation of translation	
			A5A4L5 Cluster: Avenin-like protein - Nutrient reservoir activity	
Tae-miR2003	2	2	Q5MFP8 Cluster: Low molecular weight glutenin - Nutrient reservoir activity	Q0Z7Z4 Cluster: Mitogen-activated protein kinase kinase 6
			Q00M61 Cluster: LMW-GS P-11	Q8RVT5 Cluster: Acyl-CoA-binding protein
			P16315 Cluster: Glutenin; low molecular weight subunit PTDUCD1 precursor	Q6ERC2 Cluster: Cytidine/deoxycytidylate deaminase-like protein
miR1439	13	1	P27807 Cluster: Histone H2B.1 - DNA binding	Unknown
			Q8GRI3 Cluster: Glutathione transferase F6	
			Q6RUJ2 Cluster: Glutamine synthetase	
			Q9SWW5 Cluster: Glutathione gamma-glutamylcysteinyltransferase 1 - Phytochelatin biosynthetic pathway	
			Q5QPY8 Cluster: Glycosyltransferase	
			Q9SEW1 Cluster: Heat shock protein 70	
			A7L5U5 Cluster: MAP kinase	
			Q9FVM7 Cluster: NADPH-cytochrome P450 reductase	
			Q5UBW9 Cluster: NAR2.1	
			Q9ATQ0 Cluster: LRK14 - Ser/Thr kinase	
miR159	2	1	P27807 Cluster: Histone H2B.1	Q40867 Cluster: Heat shock protein 17.9
			Q8LL15 Cluster: Glutathione-S-transferase	Q6RZV1 Cluster: 4-nitrophenylphosphatase-like protein
				Q6ZJ08 Cluster: Monodehydroascorbate reductase
				Q94KE7 Cluster: Early light-inducible protein ELIP
				Q66PW8 Cluster: Mitochondrial 2-oxoglutarate/malate translocator
miR160	4	1	A7×9Z8 Cluster: Drought-responsive factor-like transcription factor DRFL1a	Q6K223 Cluster: Auxin response factor 8
				A0S5Z4 Cluster: Peroxidase
				Q9M4Q0 Cluster: Molybdenum cofactor biosynthesis protein Cnx1
miR5064	1	1	Q2L3U4 Cluster: FIMBRIATA-like protein	Q67VK9 Cluster: Cgi67 serine protease-like; n = 2
			A1Z1R3 Cluster: Uroporphyrinogen III synthase - tetrapyrole biosynthetic process	Q69MT7 Cluster: C2 domain-containing protein-like
				A4FPI4 Cluster: Endo-1;4-beta-glucanase
				P02334 Cluster: Protamine-2C
				O22384 Cluster: Glycine-rich protein
				Q23K01 Cluster: Myb-like DNA-binding domain containing protein
				Q40073 Cluster: Ribulose bisphosphate carboxylase/oxygenase activase A; chloroplast precursor; n = 3
				A8H0×5 Cluster: Short-chain dehydrogenase/reductase SDR
				Q5JMM1 Cluster: Auxin response factor 3; n = 1
				Q2QPU8 Cluster: F-box domain containing protein; expressed
				O64805 Cluster: T1F15.13 protein; n = 1
				Q10S22 Cluster: TPR Domain containing protein
				Q6K9T9 Cluster: Metallo-beta-lactamase-like
miR5180	1	1	Unknown	Unknown
miR5200	1	1	Q3ZPM9 Cluster: Flowering locus T	
miR528	1	1	Q50HV8 Cluster: Glycosyltransferase	Unknown
			O24400 Cluster: Superoxide dismutase [Cu-Zn]	
			Q8H0B8 Cluster: Cold regulated protein	
Tae-miR2006b	1	1	Unknown	Q8RVT5 Cluster: Acyl-CoA-binding protein
				Q0Z7Z4 Cluster: Mitogen-activated protein kinase kinase 6
				Q6ERC2 Cluster: Cytidine/deoxycytidylate deaminase-like protein
Tae-miR394b	1	1	Q41524 Cluster: DNA-directed RNA polymerase	Unknown
Tae-miR818	1	1	A9J204 Cluster: MIKC-type MADS-box transcription factor WM22A	A3FKJ8 Cluster: PMCA-type calcium ATPase A2
			Q6VTH8 Cluster: Resistance-related receptor-like kinase	Q6YYS2 Cluster: Leucine zipper protein-like
				Q84XQ5 Cluster: Glyoxalase II
				Q1IWV5 Cluster: Transcriptional regulator
				A3RD20 Cluster: C-8
				7-sterol isomerase
				Q9ATV7 Cluster: Arabinoxylan arabinofuranohydrolase isoenzyme AXAH-II
				A6N022 Cluster: S-adenosylmethionine decarboxylase proenzyme
				A2QWV2 Cluster: Function: co-expression of het-e and het-c lead to cell death
				Q69LK8 Cluster: Serine protease-like protein
				Q2QPU8 Cluster: F-box domain containing protein
				Q2QP88 Cluster: CHY zinc finger family protein
				UPI000069F7AB Cluster: Phosphate-regulating neutral endopeptidase
				A6V4A8 Cluster: Probable radical activating enzyme
				Q2R078 Cluster: Leucine Rich Repeat family protein
				Q84UQ1 Cluster: DEAD-box ATP-dependent RNA helicase 42
				A3QQM8 Cluster: 12-oxophytodienoic acid reductase 1
Tae-miR2041b	1	1	Unknown	Q9ST55 Cluster: E2F protein - DNA binding
