# Supplementary material for: Deep sequencing of wheat sRNA transcriptome reveals distinct temporal expression pattern of miRNAs in response to heat, light and UV
Source: Sci Rep. 2016 Dec 22;6:39373. doi: 10.1038/srep39373 (PMC5177929; doi:10.1038/srep39373)
Supplement: Supplementary Information [file srep39373-s1.pdf]

Deep sequencing of wheat sRNA transcriptome reveals distinct temporal expression pattern of miRNAs in response to heat, light and UV

Raja Ragupathy, Sridhar Ravichandran, Md. Safiur Rahman Mahdi, Douglas Huang, Elsa Reimer, Michael Domaratzki and Sylvie Cloutier

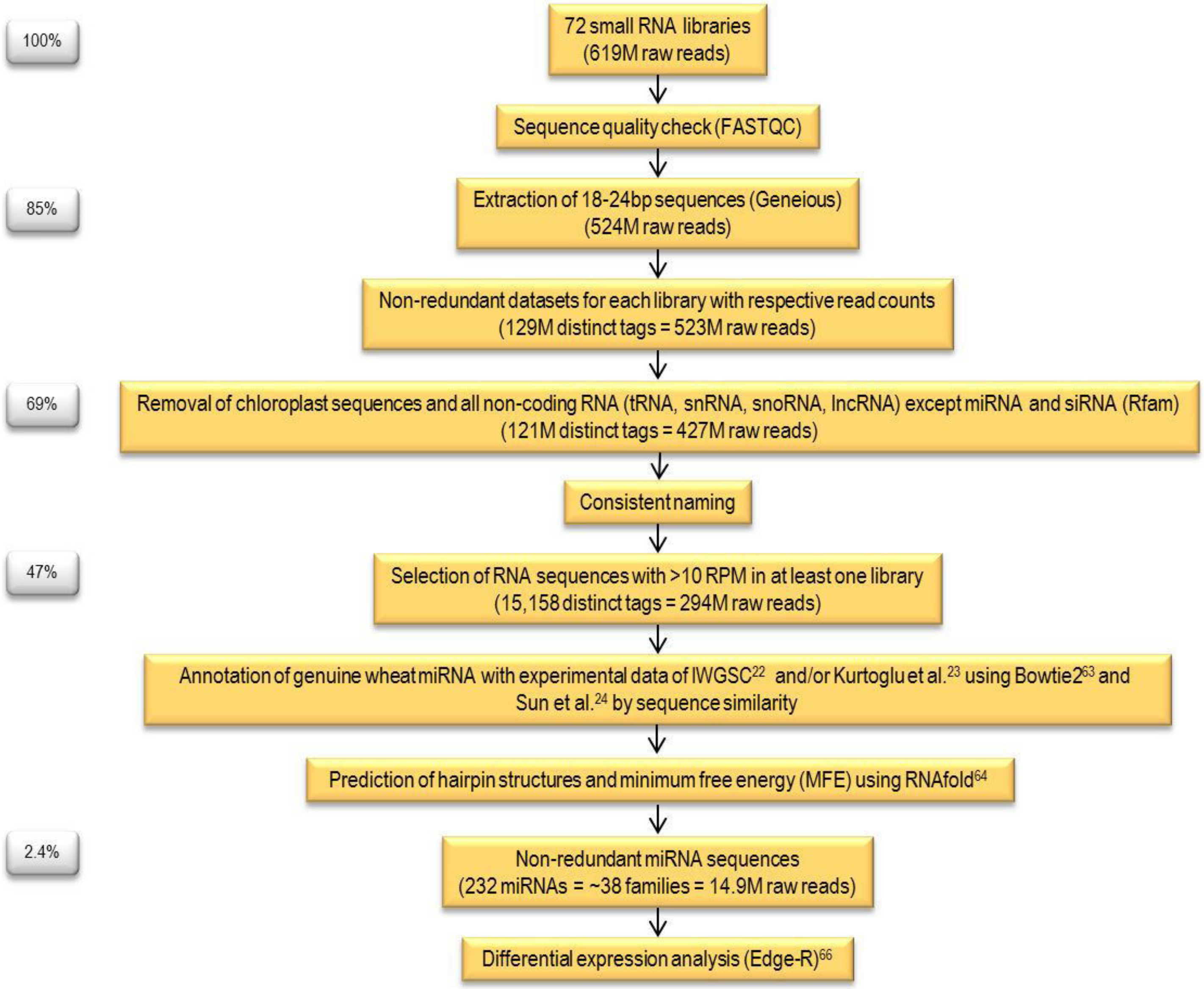

Figure S1. Processing workflow of wheat miRNAs. Raw read and distinct tag numbers are in parentheses.

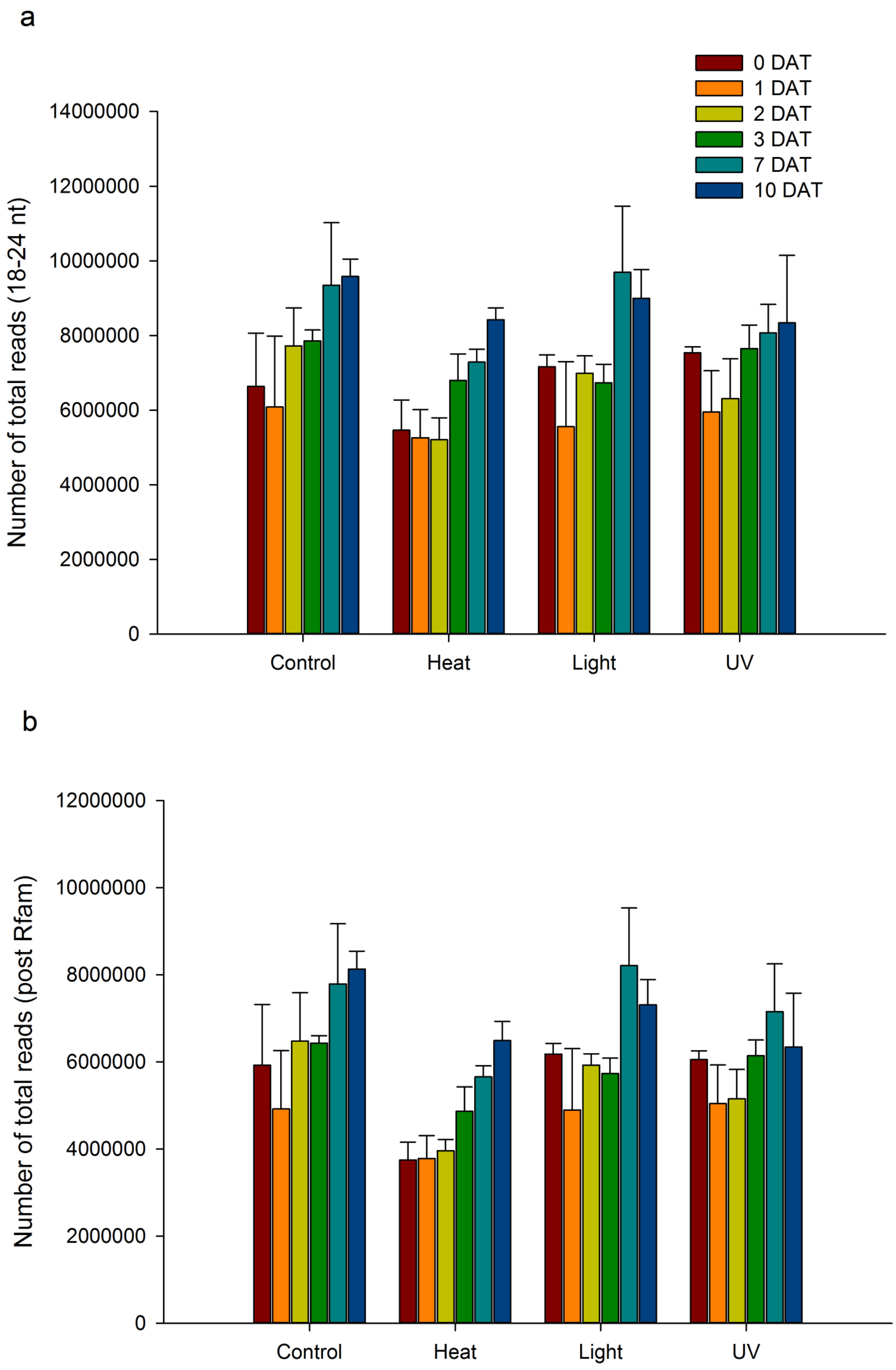

Figure S2. Overview of small RNA profile. (a) Total number of reads per treatment. **(b)** Total number of reads after removal of alternate types of non-coding RNA and chloroplast sequences.The bars represent the mean and standard deviation from three biological replicates.

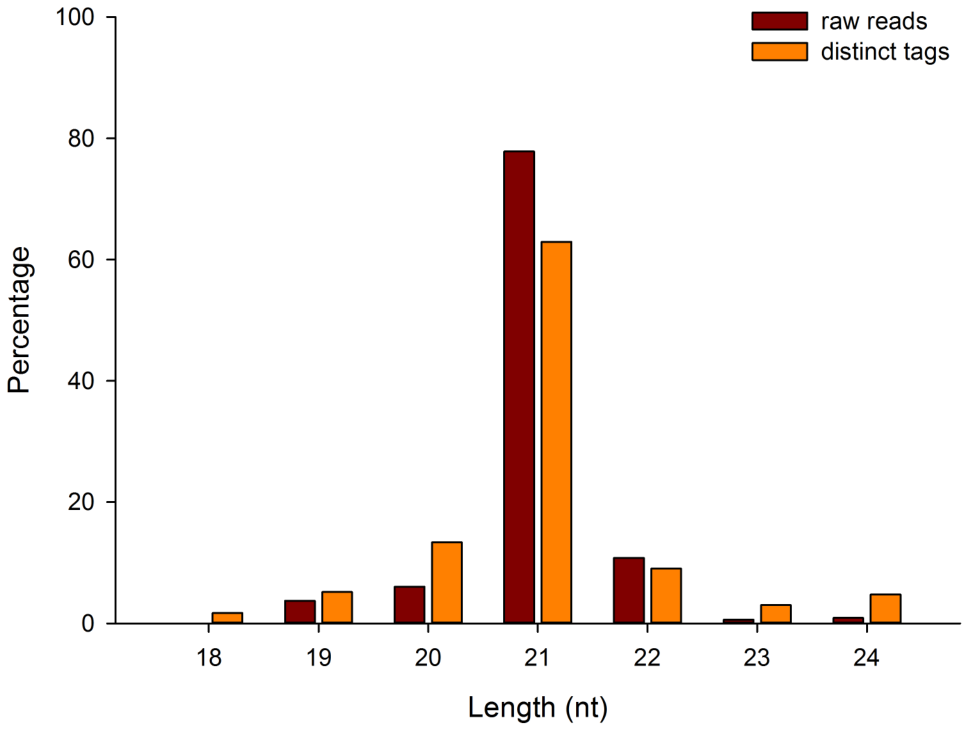

Figure S3. Size distribution of conserved miRNA families represented as percentage of raw reads and distinct tags.

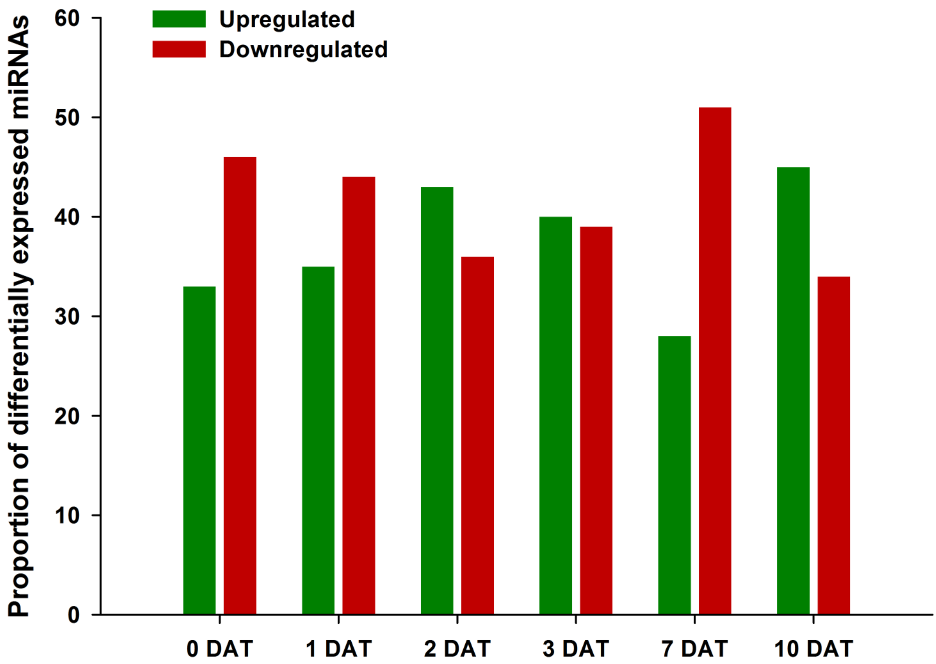

Figure S4. Proportion of differentially expressed miRNAs that were up or downregulated in response to heat stress at each sampling time point expressed as days after treatment (DAT).

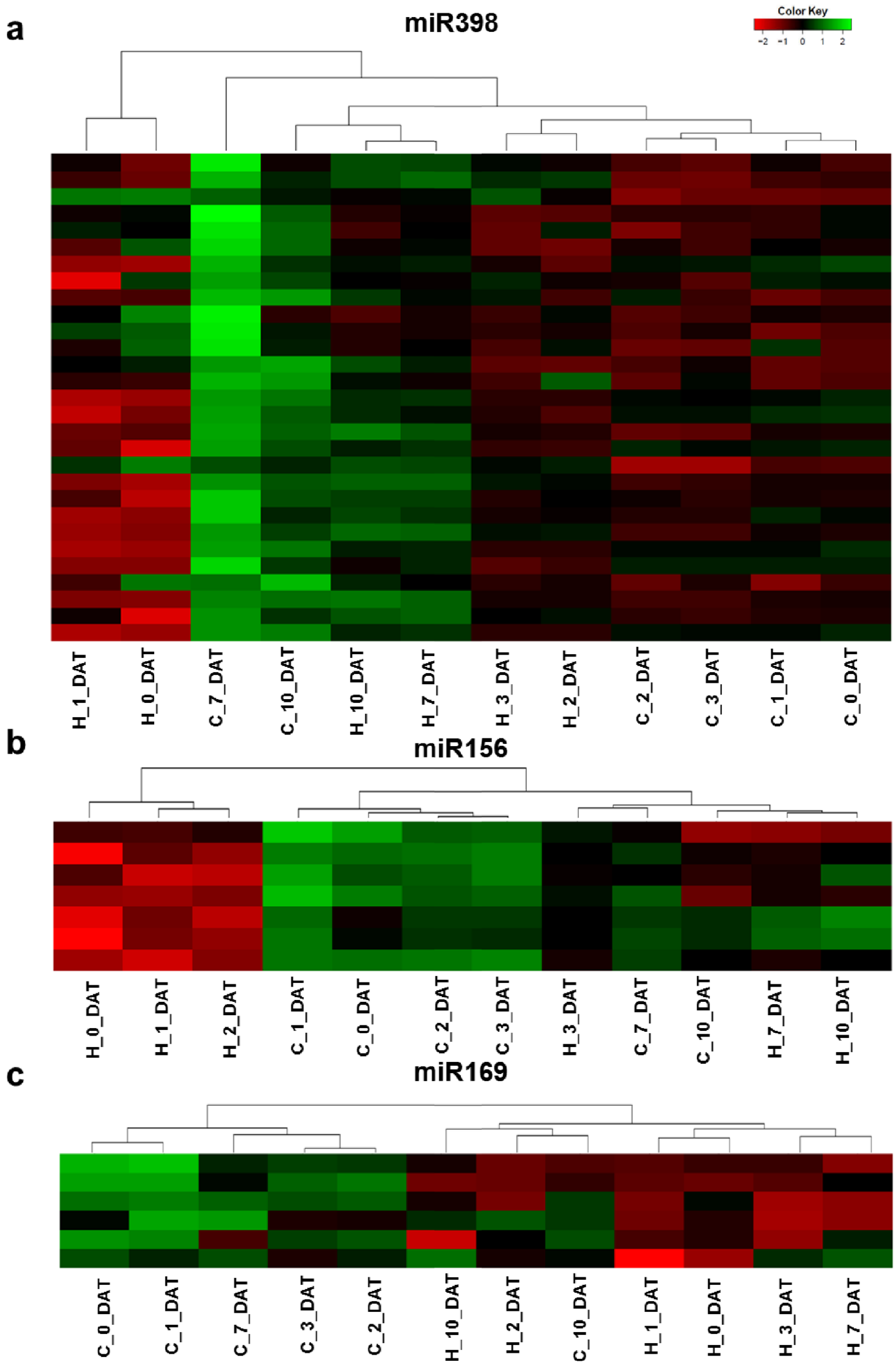
a
miR398
Color Key
-2 -1 0 1 2
H_1_DAT
H_0_DAT
C_7_DAT
C_10_DAT
H_10_DAT
H_7_DAT
H_3_DAT
H_2_DAT
C_2_DAT
C_3_DAT
C_1_DAT
C_0_DAT
b
miR156
H_0_DAT
H_1_DAT
H_2_DAT
C_1_DAT
C_0_DAT
C_2_DAT
C_3_DAT
H_3_DAT
C_7_DAT
C_10_DAT
H_7_DAT
H_10_DAT
c
miR169
C_0_DAT
C_1_DAT
C_7_DAT
C_3_DAT
C_2_DAT
H_10_DAT
H_2_DAT
C_10_DAT
H_1_DAT
H_0_DAT
H_3_DAT
H_7_DAT

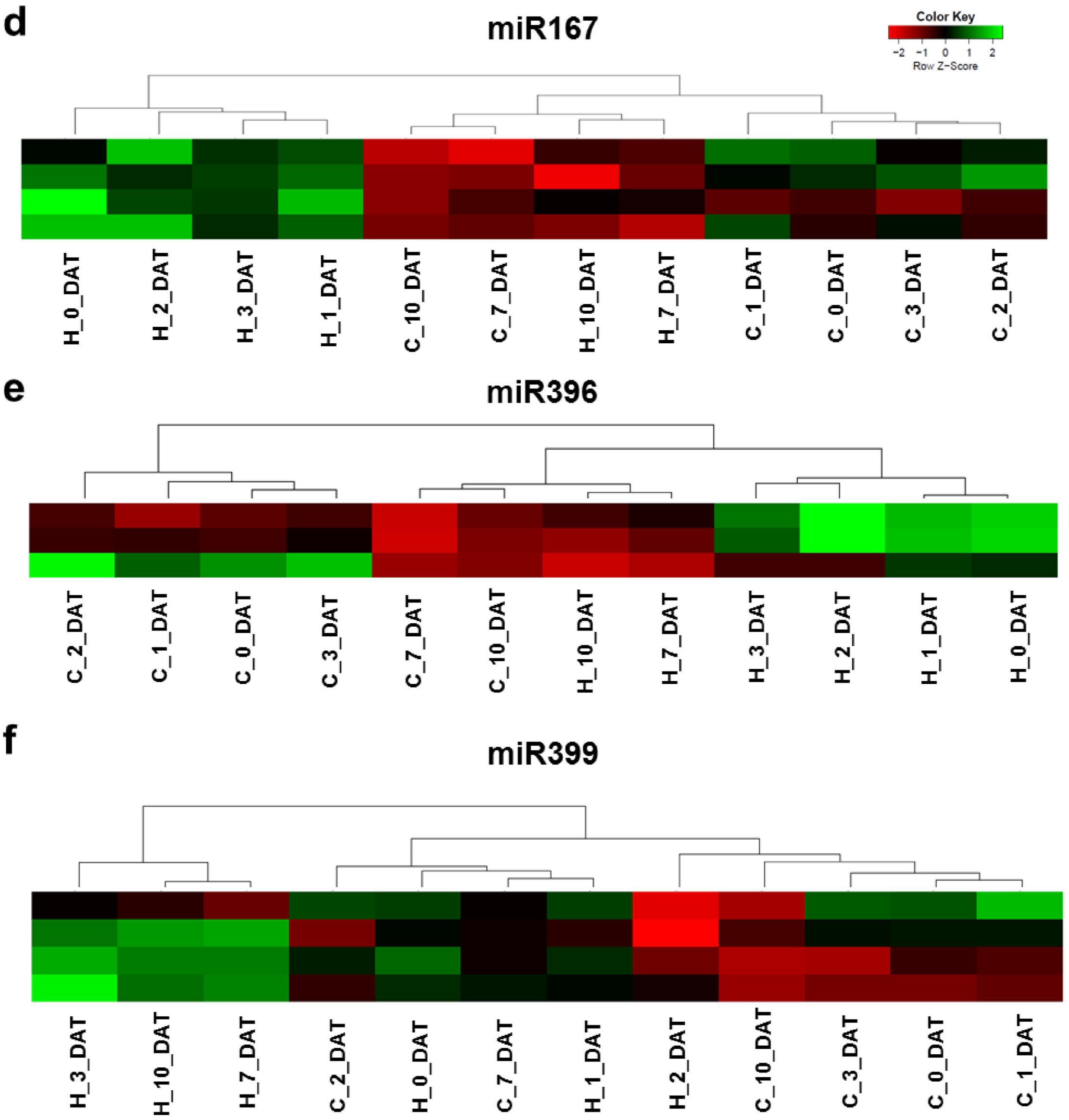

Figure S5. Heat maps of miRNA families where multiple family members and isomiRs were differentially expressed. Heat maps were generated with the moderated log2 reads per million values using R (n=3). Green represents higher expression levels than the mean while red represents lower. The dendrogram at the top represents the clustering of the control (C) and heat (H) treatments at the six sampling time points of 0, 1, 2, 3, 7 and 10 days after treatment (DAT) labeled at the bottom. **(a)** miR398, **(b**) miR156, **(c)** miR169, **(d)** miR167, **(e)** miR396, **(f)** mir399.

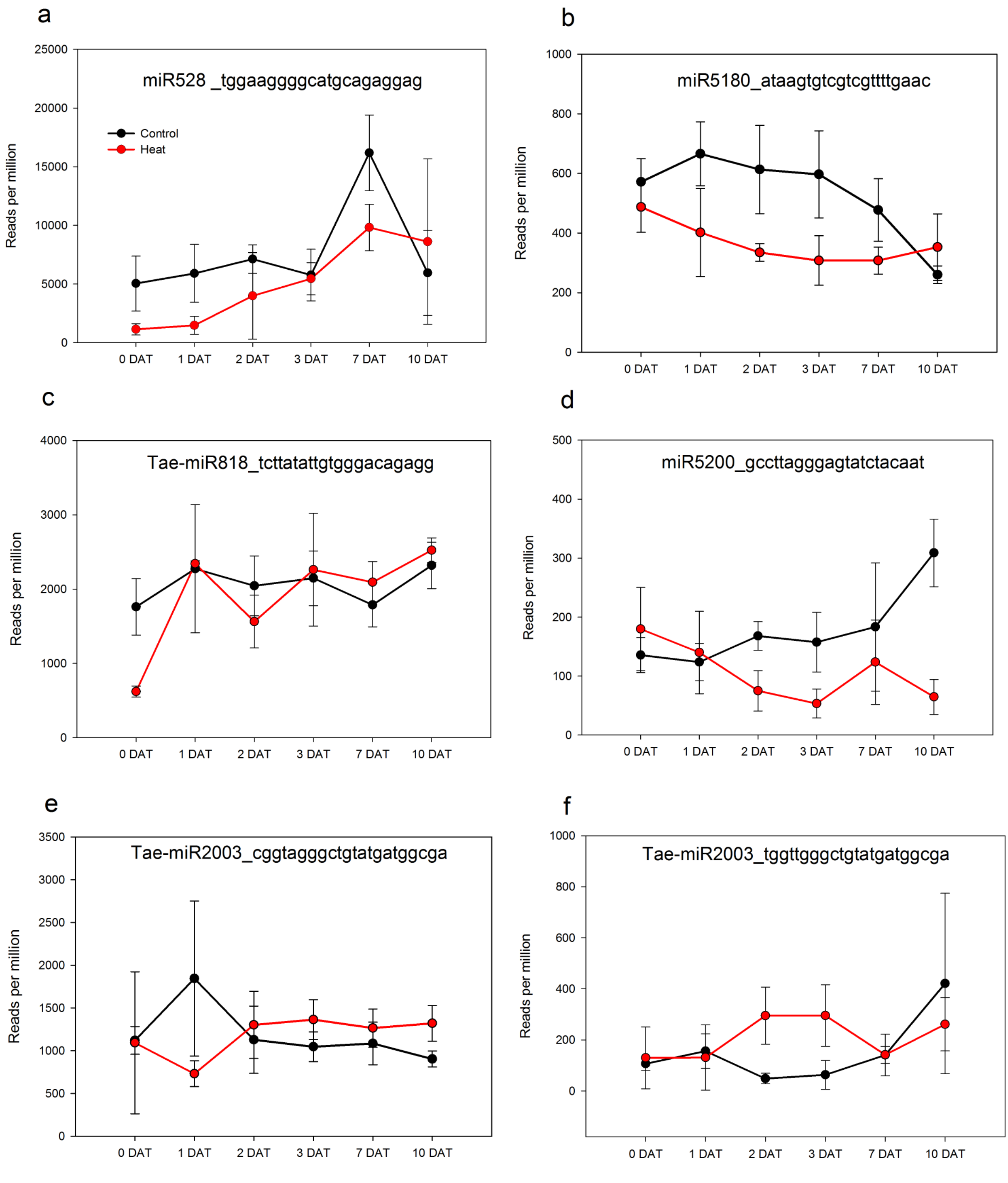
a
miR528 _tggaaggggcatgcagaggag
Control
Heat
Reads per million
25000
20000
15000
10000
5000
0
0 DAT
1 DAT
2 DAT
3 DAT
7 DAT
10 DAT
b
miR5180_ataagtgtcgtcgttttgaac
Reads per million
1000
800
600
400
200
0
0 DAT
1 DAT
2 DAT
3 DAT
7 DAT
10 DAT
c
Tae-miR818_tcttatattgtgggacagagg
Reads per million
4000
3000
2000
1000
0
0 DAT
1 DAT
2 DAT
3 DAT
7 DAT
10 DAT
d
miR5200_gccttagggagtatctacaat
Reads per million
500
400
300
200
100
0
0 DAT
1 DAT
2 DAT
3 DAT
7 DAT
10 DAT
e
Tae-miR2003_cggtagggctgtatgatggcga
Reads per million
3500
3000
2500
2000
1500
1000
500
0
0 DAT
1 DAT
2 DAT
3 DAT
7 DAT
10 DAT
f
Tae-miR2003_tggttgggctgtatgatggcga
Reads per million
1000
800
600
400
200
0
0 DAT
1 DAT
2 DAT
3 DAT
7 DAT
10 DAT

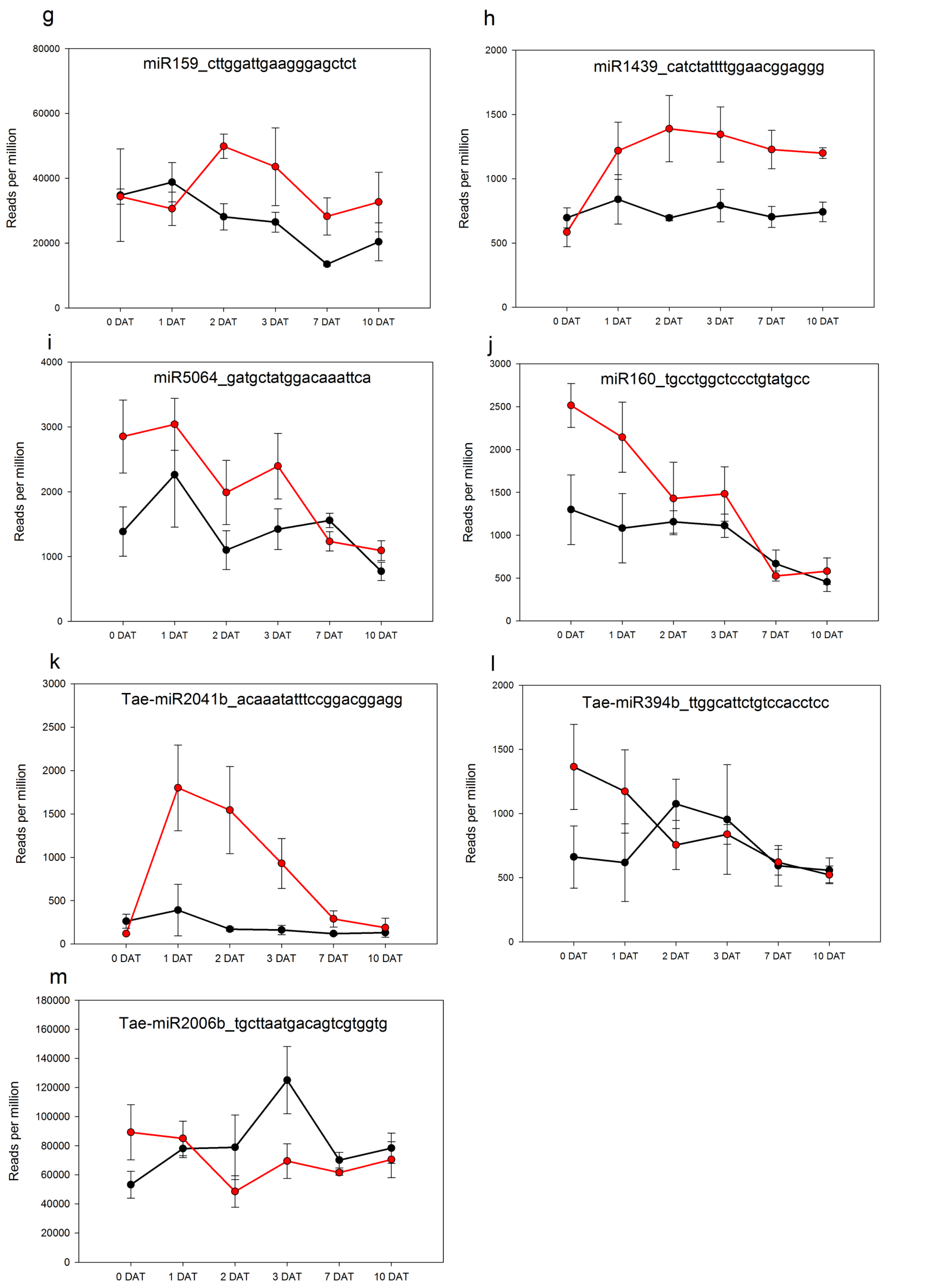

Figure S6. Line graph showing the relative abundance of miRNAs normalized as reads per million (Heat vs Control). **(a-m)** Various miRNAs as labeled in each box. The lines represent the mean and standrard deviation from three bilogical replicates.

Table S1. Sequencing and processing summary of the 72 small RNA libraries. Raw reads, distinct tags and distinct miRNAs are displayed.

						Non-redundant dataset	Removal of chloroplast and other non-coding RNAs		Distinct tags with ≥ 10 RPM in at least one library		Non-redundant miRNA sequence dataset	
Small RNA library	Treatment	Sampling time (Days after end of treatment)	Repli-cate	Raw reads	High quality reads (18-24bp)	Distinct tags	Raw reads	Distinct tags	Raw reads	Distinct tags	Raw reads	Distinct miRNAs
1	Control	0	R1	8,892,384	7,954,051	1,862,962	7,173,885	1,784,898	4,907,760	14,949	212,092	231
2	Control	0	R2	7,670,492	6,834,706	1,721,867	6,174,780	1,650,928	4,165,611	15,128	161,938	232
3	Control	0	R3	6,075,679	5,125,089	1,552,617	4,414,626	1,483,665	2,810,221	15,158	120,657	232
4	Control	1	R1	4,947,853	4,062,658	1,118,928	3,413,877	1,052,664	2,248,664	15,024	76,110	231
5	Control	1	R2	9,006,880	7,822,886	1,790,917	5,970,935	1,763,050	4,552,644	15,083	154,274	231
6	Control	1	R3	7,638,768	6,380,378	1,801,870	5,374,745	1,708,302	3,420,786	15,148	127,534	232
7	Control	2	R1	9,527,477	8,572,489	2,309,743	7,577,702	2,220,726	4,780,863	15,157	216,870	232
8	Control	2	R2	9,124,121	7,996,889	1,998,530	6,501,971	1,879,007	4,420,763	15,158	170,394	232
9	Control	2	R3	7,589,286	6,599,840	1,705,219	5,355,872	1,602,598	3,622,252	15,158	134,976	232
10	Control	3	R1	9,304,664	7,967,083	1,931,237	6,461,202	1,804,081	4,370,120	15,158	146,510	232
11	Control	3	R2	8,524,964	7,516,858	2,017,209	6,250,289	1,918,814	4,181,251	15,158	171,817	232
12	Control	3	R3	9,149,135	8,070,714	2,239,014	6,582,192	2,122,930	4,218,839	15,023	179,163	231
13	Control	7	R1	12,287,577	10,527,034	2,303,592	8,659,938	2,145,249	6,114,929	15,082	476,063	231
14	Control	7	R2	8,658,675	7,414,837	1,967,699	6,194,818	1,849,945	4,087,307	15,148	356,016	232
15	Control	7	R3	11,761,262	10,082,155	2,330,790	8,509,103	2,201,460	5,900,063	15,158	514,560	232
16	Control	10	R1	11,305,448	9,929,272	2,363,429	8,526,198	2,238,476	5,783,145	15,158	374,207	232
17	Control	10	R2	11,088,839	9,761,978	2,423,642	8,160,435	2,306,961	5,512,478	15,158	388,610	232
18	Control	10	R3	10,361,530	9,052,566	2,172,642	7,708,991	2,063,041	5,158,765	15,158	217,437	232
19	Heat	0	R1	5,775,948	4,544,251	1,036,511	3,325,346	929,225	2,390,236	15,147	41,548	232
20	Heat	0	R2	7,271,220	5,783,423	1,112,982	3,774,830	976,523	2,889,869	15,158	43,212	232
21	Heat	0	R3	7,706,788	6,064,609	1,254,552	4,142,773	1,121,602	3,042,845	14,494	56,038	215
22	Heat	1	R1	6,740,906	5,084,398	1,043,633	3,432,600	943,236	2,775,388	15,041	47,944	231
23	Heat	1	R2	7,748,601	6,090,675	1,251,622	4,386,462	1,137,191	3,522,358	15,103	61,888	231
24	Heat	1	R3	6,302,025	4,613,125	995,059	3,522,377	908,062	2,737,701	15,150	42,490	232

						Non-redundant dataset	Removal of chloroplast and other non-coding RNAs		Distinct tags with ≥ 10 RPM in at least one library		Non-redundant miRNA sequence dataset	
Small RNA library	Treatment	Sampling time (Days after end of treatment)	Repli-cate	Raw reads	High quality reads (18-24bp)	Distinct tags	Raw reads	Distinct tags	Raw reads	Distinct tags	Raw reads	Distinct miRNAs
25	Heat	2	R1	7,153,609	5,872,789	1,463,322	4,258,188	1,360,102	2,919,069	15,157	92,875	232
26	Heat	2	R2	6,490,161	4,988,186	1,268,978	3,780,938	1,169,145	2,725,760	15,158	73,844	232
27	Heat	2	R3	6,344,509	4,764,107	1,271,939	3,835,718	1,184,748	2,697,291	15,158	90,597	232
28	Heat	3	R1	7,882,221	6,069,544	1,536,225	4,228,876	1,401,367	3,069,561	15,158	81,265	232
29	Heat	3	R2	8,560,115	6,831,434	1,855,547	5,200,461	1,753,594	3,741,099	14,760	110,818	228
30	Heat	3	R3	9,461,260	7,488,938	1,932,095	5,179,769	1,777,766	3,721,519	15,037	101,772	231
31	Heat	7	R1	9,127,311	7,497,557	1,726,070	5,482,441	1,591,398	4,066,902	15,102	157,752	231
32	Heat	7	R2	8,083,858	6,886,546	1,792,722	5,540,090	1,682,756	3,774,501	15,150	193,298	232
33	Heat	7	R3	9,174,255	7,475,776	1,991,955	5,945,687	1,856,948	3,981,000	15,157	193,128	232
34	Heat	10	R1	11,130,599	8,788,873	1,977,247	6,069,775	1,781,871	4,565,798	15,158	187,060	232
35	Heat	10	R2	9,744,300	8,247,600	2,125,387	6,949,395	2,022,950	4,665,009	15,158	159,389	232
36	Heat	10	R3	9,516,161	8,236,354	2,036,309	6,445,620	1,906,077	4,537,450	15,158	187,937	232
37	Light	0	R1	8,467,794	7,367,439	2,089,019	6,395,355	1,998,186	4,080,181	15,158	189,789	232
38	Light	0	R2	8,069,271	6,807,080	1,801,813	5,913,486	1,590,127	4,130,930	15,158	218,617	232
39	Light	0	R3	8,421,439	7,324,748	1,950,603	6,230,547	1,858,932	4,139,243	14,972	236,968	231
40	Light	1	R1	6,397,582	5,452,030	1,443,266	5,399,009	1,364,302	3,089,401	15,051	120,864	231
41	Light	1	R2	8,744,036	7,351,130	1,726,653	5,982,678	1,609,165	4,270,164	15,128	161,881	232
42	Light	1	R3	4,511,488	3,866,319	953,658	3,302,661	891,141	2,354,269	15,152	84,014	232
43	Light	2	R1	8,819,854	7,522,036	1,866,087	6,227,094	1,754,918	4,463,845	15,157	173,807	232
44	Light	2	R2	7,650,686	6,671,291	1,673,909	5,778,573	1,584,884	3,971,510	15,158	174,817	232
45	Light	2	R3	7,505,311	6,773,196	1,681,601	5,766,249	1,587,905	3,942,928	15,158	199,812	232
46	Light	3	R1	7,201,833	6,383,381	1,674,521	5,522,058	1,600,482	3,654,600	15,158	157,410	232
47	Light	3	R2	7,279,631	6,515,602	1,574,651	5,540,321	1,483,986	3,792,449	14,962	149,862	231
48	Light	3	R3	8,430,957	7,299,915	1,729,866	6,139,804	1,619,909	4,284,040	15,049	150,784	231
49	Light	7	R1	11,554,207	10,252,126	2,324,072	8,429,005	2,189,951	5,908,583	15,143	759,854	232
50	Light	7	R2	12,655,425	11,116,456	2,486,979	9,410,418	2,347,545	6,543,217	15,152	781,442	232

						Non-redundant dataset	Removal of chloroplast and other non-coding RNAs		Distinct tags with ≥ 10 RPM in at least one library		Non-redundant miRNA sequence dataset	
Small RNA library	Treatment	Sampling time (Days after end of treatment)	Repli-cate	Raw reads	High quality reads (18-24bp)	Distinct tags	Raw reads	Distinct tags	Raw reads	Distinct tags	Raw reads	Distinct miRNAs
51	Light	7	R3	8,716,499	7,699,841	1,967,116	6,788,543	1,864,283	4,560,957	15,157	503,169	232
52	Light	10	R1	9,574,366	8,149,714	2,159,360	6,671,378	2,043,978	4,436,346	15,158	397,433	232
53	Light	10	R2	10,551,777	9,177,379	2,276,314	7,451,969	2,144,217	5,067,940	15,158	409,997	232
54	Light	10	R3	11,452,465	9,657,322	2,335,165	7,805,561	2,176,176	5,354,305	15,158	425,044	232
55	UV	0	R1	8,868,311	7,498,533	1,882,176	6,237,119	1,769,830	4,233,601	15,158	138,846	232
56	UV	0	R2	9,434,413	7,713,193	1,801,813	6,079,291	1,672,185	4,203,815	15,158	117,755	232
57	UV	0	R3	8,700,302	7,401,219	1,681,419	5,834,165	1,559,557	4,251,958	15,020	143,315	231
58	UV	1	R1	5,816,220	4,978,405	1,119,864	4,309,137	1,055,256	3,178,544	15,072	94,427	232
59	UV	1	R2	8,607,392	7,152,749	1,861,112	6,028,712	1,763,050	4,072,677	15,144	170,427	232
60	UV	1	R3	6,819,454	5,723,798	1,576,116	4,782,552	1,488,834	3,203,764	15,152	113,666	232
61	UV	2	R1	8,599,918	7,165,963	1,821,007	5,479,305	1,691,786	3,937,994	15,157	141,379	232
62	UV	2	R2	6,016,954	5,113,993	1,214,715	4,380,064	1,137,997	3,189,427	15,158	107,528	232
63	UV	2	R3	7,763,886	6,656,062	1,684,686	5,601,662	1,582,171	3,894,111	15,158	127,201	232
64	UV	3	R1	8,132,061	6,922,110	1,832,705	5,739,177	1,727,474	3,868,742	15,158	131,588	232
65	UV	3	R2	9,622,805	8,073,855	2,251,912	6,233,490	2,136,281	4,284,155	13,604	189,051	231
66	UV	3	R3	9,234,189	7,948,992	2,145,919	6,444,604	2,024,344	4,275,776	15,066	176,408	231
67	UV	7	R1	9,898,899	8,490,916	2,030,865	8,213,656	1,902,893	4,793,669	15,147	376,387	232
68	UV	7	R2	9,843,084	8,532,772	1,980,148	7,218,307	1,853,057	5,050,203	15,153	371,101	232
69	UV	7	R3	8,312,251	7,191,818	1,633,590	6,018,565	1,516,294	4,324,990	15,157	375,970	232
70	UV	10	R1	9,232,488	7,672,946	1,777,350	5,906,077	1,635,542	4,293,326	15,158	249,699	232
71	UV	10	R2	8,314,249	6,961,867	1,684,655	5,381,714	1,548,006	3,778,289	15,158	173,789	232
72	UV	10	R3	12,761,965	10,384,851	2,403,684	7,738,621	2,218,775	5,415,164	15,158	243,559	232
**Total or [Max]**				**619,112,343**	**523,970,715**	**129,382,451**	**426,893,832**	**121,294,775**	**294,374,930**	**[15,158]**	**14,859,743**	**[232]**

Tables S2 and S3 are submitted as Excel spreedsheet

Table S4. Molecular function classification of predicted miRNA targets.

Gene ontology identifier	Molecular function	Number of targets
GO:0001071	nucleic acid binding transcription factor activity	5
GO:0003677	DNA binding	13
GO:0003723	RNA binding	3
GO:0004386	helicase activity	1
GO:0004871	signal transducer activity	2
GO:0008135	translation factor activity, RNA binding	2
GO:0008233	peptidase activity	2
GO:0008289	lipid binding	1
GO:0016301	kinase activity	6
GO:0016491	oxidoreductase activity	6
GO:0016746	transferase activity, transferring acyl groups	1
GO:0016757	transferase activity, transferring glycosyl groups	2
GO:0016765	transferase activity, transferring alkyl or aryl (other than methyl) groups	1
GO:0016779	nucleotidyltransferase activity	1
GO:0016798	hydrolase activity, acting on glycosyl bonds	2
GO:0016829	lyase activity	2
GO:0016853	isomerase activity	2
GO:0016874	ligase activity	1
GO:0016887	ATPase activity	1
GO:0019899	enzyme binding	1
GO:0043167	ion binding	26

Table S5. List of predicted interaction partners identified in maize (*Zea mays*) using STRING DB.

Protein	Description
MPK7	ABA stimulation MAP kinase (398 aa)
chiB	Endochitinase B Precursor; Fragment (EC 3.2.1.14)(Seed chitinase B); Defense against chitin containing fungal pathogens (281 aa)
GRMZM2G019863_P01	flowering locus D (808 aa)
gs1-2	Glutamine synthetase root isozyme 2 (EC 6.3.1.2)(Glutamate--ammonia ligase); Plays a role in the flow of nitrogen into nitrogenous organic compounds (369 aa)
SODCC.1	Superoxide dismutase [Cu-Zn] 2 (EC 1.15.1.1); Destroys radicals which are normally produced within the cells and which are toxic to biological systems (By similarity) (210 aa)
hsp70-5	Heat shock protein 70 Fragment (649 aa)
L-FNRII	ferredoxin (368 aa)
SODA.4	Superoxide dismutase [Mn] 3.1, mitochondrial Precursor (EC 1.15.1.1); Destroys superoxide anion radicals which are normally produced within the cells and which are toxic to biological systems (235 aa)
GRMZM2G062552_P01	glycosyltransferase (488 aa)
histone2b1	Histone H2B.1 ; Core component of nucleosome. Nucleosomes wrap and compact DNA into chromatin, limiting DNA accessibility to the cellular machineries which require DNA as a template. Histones thereby play a central role in transcription regulation, DNA repair, DNA replication and chromosomal stability. DNA accessibility is regulated via a complex set of post-translational modifications of histones, also called histone code, and nucleosome remodeling (By similarity) (151 aa)
CRS1	Chloroplastic group IIA intron splicing facilitator CRS1, chloroplastic Precursor (Protein CHLOROPLAST RNA SPLICING 1)(Chloroplastic RNA splicing factor 1); Required for the splicing of group IIA introns in chloroplasts, and especially for atpF, by regulating the intron folding. Forms splicing particles with RNA. Also involved in chloroplast protein translation (715 aa)
INO1	Inositol-3-phosphate synthase (MIP synthase)(EC 5.5.1.4)(Myo-inositol-1-phosphate synthase)(MI-1-P synthase)(IPS) (510 aa)
PIP1-1	Aquaporin PIP1-1 (Plasma membrane intrinsic protein 1-1)(ZmPIP1-1)(ZmPIP1;1)(ZmPIP1a); Water channel required to facilitate the transport of water across cell membrane. Active as heteromers with PIP1-2, but not as homomers (288 aa)
NAR2.1	high affinity nitrate transporter (203 aa)
rpoC1	DNA-directed RNA polymerase subunit beta' (EC 2.7.7.6)(PEP)(Plastid-encoded RNA polymerase subunit beta')(RNA polymerase subunit beta'); DNA-dependent RNA polymerase catalyzes the transcription of DNA into RNA using the four ribonucleoside triphosphates as substrates (683 aa)
rpoB	DNA-directed RNA polymerase subunit beta (EC 2.7.7.6)(PEP)(Plastid-encoded RNA polymerase subun [...] (1075 aa)
GLSF	Ferredoxin-dependent glutamate synthase, chloroplastic Precursor (EC 1.4.7.1)(Fd-GOGAT) (1616 aa)
rpoC2	DNA-directed RNA polymerase subunit beta'' (EC 2.7.7.6)(PEP)(Plastid-encoded RNA polymerase sub [...] (1527 aa)
RPD2	RNA polymerase IV second largest subunit (1229 aa)
GRMZM2G133512_P01	DNA-directed RNA polymerase (1259 aa)
GLSF	Ferredoxin-dependent glutamate synthase, chloroplastic Precursor (EC 1.4.7.1)(Fd-GOGAT) (1616 aa)
GRMZM2G468367_P01	annotation not available (492 aa)

Protein	Description
GRMZM2G126913_P02	annotation not available (762 aa)
GRMZM5G895064_P01	DNA-directed RNA polymerase (EC 2.7.7.6) (362 aa)
GRMZM5G881057_P01	annotation not available (57 aa)
rpoB	DNA-directed RNA polymerase subunit beta (EC 2.7.7.6)(PEP)(Plastid-encoded RNA polymerase subunit beta)(RNA polymerase subunit beta); DNA-dependent RNA polymerase catalyzes the transcription of DNA into RNA using the four ribonucleoside triphosphates as substrates (1075 aa)
GRMZM5G867785_P01	annotation not available (92 aa)
GRMZM2G456885_P01	annotation not available (309 aa)
pco108652	F-box domain containing protein (493 aa)
AC213612.3_FGP001	glycine-rich cell wall structural protein (151 aa)
not1	Neighbor of TGA1 (588 aa)
pco075539	hypothetical protein LOC100274472 (461 aa)
GRMZM2G040513_P01	TPR domain containing protein (253 aa)
pco142465	hypothetical protein LOC100192021 (241 aa)
GRMZM2G055705_P01	Leucine Rich Repeat family protein (233 aa)
GRMZM2G056270_P01	RING finger and CHY zinc finger domain-containing protein 1 (293 aa)
GRMZM2G060369_P01	Putative uncharacterized protein (392 aa)
GRMZM2G065194_P01	short-chain dehydrogenase/reductase SDR (341 aa)
GRMZM2G079908_P01	Acyl-CoA-binding proteinPutative uncharacterized protein ; (116 aa)
GRP	glycine-rich protein1 (155 aa)
GRMZM2G099101_P01	endo-1,4-beta-glucanase (620 aa)
cytP450	Putative uncharacterized protein (536 aa)
GRMZM2G120408_P01	Putative uncharacterized protein (418 aa)
pox3	Peroxidase Fragment (360 aa)
GRMZM2G154839_P01	regulator of telomere elongation helicase 1 (941 aa)
x1	hypothetical protein LOC100279850 (628 aa)
elip2	early light-inducible protein ELIP (LOC541918), mRNA (186 aa)
psaC	Photosystem I iron-sulfur center (Photosystem I subunit VII)(9 kDa polypeptide)(PSI-C)(PsaC); Apoprotein for the two 4Fe-4S centers FA and FB of photosystem I (PSI); essential for photochemical activity. FB is the terminal electron acceptor of PSI, donating electrons to ferredoxin. The C-terminus interacts with PsaA/B/D and helps assemble the protein into the PSI complex. Required for binding of PsaD and PsaE to PSI. PSI is a plastocyanin-ferredoxin oxidoreductase, converting photonic excitation into a charge separation, which transfers an electron from the donor P700 chlorophyll pair [...] (81 aa)
psi-d1	Photosystem I reaction center subunit II (236 aa)
GRMZM2G013342_P01	hypothetical protein LOC100191984 (199 aa)
GRMZM2G017290_P01	photosystem I reaction center subunit III (225 aa)

Protein	Description
GRMZM2G085646_P01	annotation not available (232 aa)
GRMZM2G473891_P01	annotation not available (612 aa)

Table S6. List of predicted interaction partners identified in rice (*Oryza sativa*) using STRING DB.

Protein	Description
OST1B	dolichyl-diphosphooligosaccharide--protein glycosyltransferase 67 kDasubunit precursor, putative, expressed; Essential subunit of the N-oligosaccharyl transferase (OST) complex which catalyzes the transfer of a high mannose oligosaccharide from a lipid-linked oligosaccharide donor to an asparagine residue within an Asn-X-Ser/Thr consensus motif in nascent polypeptide chains (By similarity) (510 aa)
13113.m00205	wall-associated kinase-like 2, putative (316 aa)
4326102	resistance-related receptor-like kinase, putative, expressed (630 aa)
OsJ_04874	RNA polymerase IV largest subunit, putative (1570 aa)
LOC_Os02g01340.1	ferredoxin--NADP reductase, chloroplast precursor, putative, expressed (376 aa)
4330649	glutamine synthetase, catalytic domain containing protein, expressed; High-affinity glutamine synthetase. May be a major component of the cytosolic glutamine synthetic pathway in leaf blades (356 aa)
4331917	inositol-3-phosphate synthase, putative, expressed (510 aa)
4332082	copper/zinc superoxide dismutase, putative, expressed; Destroys radicals which are normally produced within the cells and which are toxic to biological systems (By similarity) (164 aa)
4332420	DnaK family protein, putative, expressed (653 aa)
H2B1	Core histone H2A/H2B/H3/H4 domain containing protein, putative; Core component of nucleosome. Nucleosomes wrap and compact DNA into chromatin, limiting DNA accessibility to the cellular machineries which require DNA as a template. Histones thereby play a central role in transcription regulation, DNA repair, DNA replication and chromosomal stability. DNA accessibility is regulated via a complex set of post-translational modifications of histones, also called histone code, and nucleosome remodeling (417 aa)
4332475	CGMC_MAPKCGMC_2_ERK.2 - CGMC includes CDA, MAPK, GSK3, and CLKC kinases, expressed; Involved in disease resistance and abiotic stress tolerance signaling pathways. Acts as a positive regulator of drought, salt and cold tolerance. Negatively modulates pathogenesis-related (PR) gene expression and broad-spectrum disease resistance (369 aa)
4338417	superoxide dismutase, mitochondrial precursor, putative, expressed; Destroys superoxide anion radicals which are normally produced within the cells and which are toxic to biological systems (231 aa)
4340184	osFTL3 FT-Like3 homologous to Flowering Locus T gene; contains Pfam profile PF01161- Phosphatidylethanolamine-binding protein, expressed; Probable mobile flower-promoting signal (florigen) that moves from the leaf to the shoot apical meristem (SAM) and induces flowering. Promotes the transition from vegetative growth to flowering under long day (LD) conditions. Acts upstream of MADS14 and MADS15. May also participate in the promotion of flowering under short day (SD) conditions (178 aa)
4342593	thioredoxin, putative, expressed; Thiol-disulfide oxidoreductase involved in the redox regulation of MAP kinases. Under reducing conditions, inhibits MPK1 and MPK5 kinase activities. Mediates its own transport from cell-to-cell through plasmodesmata. Possesses insulin disulfide bonds reducing activity (122 aa)
OsJ_03025	glutamate synthase, chloroplast precursor, putative, expressed; Involved in glutamate biosynthesis and plays a major role in the primary ammonium ions assimilation in seedling roots. May be involved in the reutilization of glutamine in developing organs. Plays a role in the development of tillers (2167 aa)

Protein	Description
3131432	RNA polymerase beta chain; DNA-dependent RNA polymerase catalyzes the transcription of DNA into RNA using the four ribonucleoside triphosphates as substrates (By similarity) (1075 aa)
4344774	DNA-directed RNA polymerase subunit, putative, expressed (1257 aa)
LOC_Os05g22860.1	DNA-directed RNA polymerase subunit beta, putative (408 aa)
4338310	DNA-directed RNA polymerase subunit beta, putative (573 aa)
4337169	DNA-directed RNA polymerase subunit, putative, expressed (1252 aa)
LOC_Os04g16820.1	DNA-directed RNA polymerase subunit beta, putative, expressed (1067 aa)
4333571	DNA-directed RNA polymerase II subunit RPB2, putative, expressed (531 aa)
4344164	ferredoxin-dependent glutamate synthase, chloroplast precursor, putative, expressed; Involved in glutamate biosynthesis in leaf. Required for the reassimilation of
4324876	dolichyl-diphosphooligosaccharide--protein glycosyltransferase 63 kDasubunit precursor, putative, expressed; Essential subunit of the N-oligosaccharyl
OsJ_00405	OsFBX1 - F-box domain containing protein, expressed (408 aa)
4324081	glycine-rich cell wall structural protein precursor, putative, expressed (124 aa)
4327698	C2 domain containing protein, putative, expressed (674 aa)
4325129	peroxidase precursor, putative, expressed (335 aa)
OsJ_01869	exo70 exocyst complex subunit family protein, putative, expressed (681 aa)
OsJ_03530	aminotransferase, classes I and II, domain containing protein, expressed; Important for the metabolism of amino acids and Krebs- cycle related organic acids. In plants, it is involved in nitrogen metabolism and in aspects of carbon and energy metabolism (460 aa)
OsJ_04096	josephin, putative, expressed; Interacts with key regulators of transcription and represses transcription. Acts as a histone-binding protein that regulates transcription. Acts as a deubiquitinating enzyme (By similarity) (336 aa)
SPL2	OsSPL2 - SBP-box gene family member, expressed; Trans-acting factor that binds specifically to the consensus nucleotide sequence 5’-TNCGTACAA-3’ (By similarity). May be involved in panicle development (412 aa)
4329556	bifunctional monodehydroascorbate reductase and carbonic anhydrasenectarin-3 precursor, putative, expressed (266 aa)
4330045	auxin response factor, putative, expressed; Auxin response factors (ARFs) are transcriptional factors that binds specifically to the DNA sequence 5’-TGTCTC-3’ found in the auxin-responsive promoter elements (AuxREs) (681 aa)
4331584	MYB family transcription factor, putative, expressed (247 aa)
4331940	OsFBX77 - F-box domain containing protein, expressed (178 aa)
4332134	cytochrome P450, putative, expressed (506 aa)
OSGRP1	RNA recognition motif containing protein, expressed (162 aa)
4333724	ankyrin, putative, expressed (541 aa)
4336294	S-adenosyl-l-methionine decarboxylase leader peptide, putative, expressed (450 aa)

Protein	Description
4337434	acyl-CoA-binding domain-containing protein 6, putative, expressed (336 aa)
4347151	expressed protein (496 aa)
4347581	metallo-beta-lactamase family protein, putative, expressed (336 aa)
LOC_Os10g39990.1	short-chain dehydrogenase/reductase SDR, putative (105 aa)
LOC_Os11g25030.1	mitochondrial 2-oxoglutarate/malate translocator, putative (54 aa)
OsJ_35885	4-nitrophenylphosphatase-like, putative, expressed (235 aa)
4334837	KAP-2, putative, expressed; Single stranded DNA-dependent ATP-dependent helicase. Involved in DNA non-homologous end joining (NHEJ) required for double-strand break repair. When associated with KU70, binds to double-stranded telomeric and non-telomeric DNA sequences, but not to single-stranded DNA. Plays a role in maintaining telomere length. Acts as a negative regulator of telomerase (By similarity) (688 aa)
4342077	heat shock protein, putative, expressed (812 aa)
4352024	telomerase reverse transcriptase, putative, expressed; Telomerase is a ribonucleoprotein enzyme essential for the replication of chromosome termini in most eukaryotes. It elongates telomeres. It is a reverse transcriptase that adds simple sequence repeats to chromosome ends by copying a template sequence within the RNA component of the enzyme (By similarity) (1259 aa)
OsJ_16077	DNA ligase I, ATP-dependent family protein, expressed (1184 aa)
4335834	expressed protein (1101 aa)
4352339	heat shock protein, putative, expressed (811 aa)
OsJ_30133	hsp90 protein, expressed (1046 aa)
LOC_Os09g30439.1	heat shock protein, putative (830 aa)
4347406	heat shock protein, putative (830 aa)
4347402	heat shock protein, putative, expressed; Molecular chaperone that promotes the maturation, structural maintenance and proper regulation of specific target proteins involved for instance in cell cycle control and signal transduction. Undergoes a functional cycle that is linked to its ATPase activity. This cycle probably induces conformational changes in the client proteins, thereby causing their activation. Interacts dynamically with various co-chaperones that modulate its substrate recognition, ATPase cycle and chaperone function (By similarity)
